# Seasonal stability of the rumen microbiome contributes to the adaptation patterns to extreme environmental conditions in grazing yak and cattle

**DOI:** 10.1186/s12915-024-02035-4

**Published:** 2024-10-23

**Authors:** Wei Guo, Mi Zhou, Fuyong Li, André Luis Alves Neves, Tao Ma, Sisi Bi, Weiwei Wang, Ruijun Long, Le Luo Guan

**Affiliations:** 1 Key Laboratory of Plateau Mountain Animal Genetics, Breeding and Reproduction, Ministry of Education, College of Animal Science, Guizhou University, Guiyang, 550025 China; 2 Department of Agricultural, Food and Nutritional Science, University of Alberta, Edmonton, AB T6G 2P5 Canada; 3 State Key Laboratory of Grassland Agro-Ecosystems, International Centre of Tibetan Plateau Ecosystem Management, School of Life Sciences, Lanzhou University, Lanzhou, 730000 China; 4 Department of Veterinary and Animal Sciences, Faculty of Health and Medical Sciences, University of Copenhagen, Grønnegårdsvej 3, Frederiksberg C, 1870 Denmark; 5 Key Laboratory of Feed Biotechnology of the Ministry of Agriculture and Rural Affairs, Institute of Feed Research, Chinese Academy of Agricultural Sciences, Beijing, 100081 China; 6 Faculty of Land and Food Systems, The University of British Columbia, Vancouver, BC V6T 1Z4 Canada; 7 Department of Animal Science and Technology, College of Animal Sciences, Zhejiang University, Hangzhou, 310058 China

**Keywords:** Rumen microbiome stability, Rumen metagenome, Microbiome functionality, Yak, Cattle

## Abstract

**Background:**

The rumen microbiome plays an essential role in maintaining ruminants’ growth and performance even under extreme environmental conditions, however, which factors influence rumen microbiome stability when ruminants are reared in such habitats throughout the year is unclear. Hence, the rumen microbiome of yak (less domesticated) and cattle (domesticated) reared on the Qinghai-Tibetan Plateau through the year were assessed to evaluate temporal changes in their composition, function, and stability.

**Results:**

Rumen fermentation characteristics and pH significantly shifted across seasons in both cattle and yak, but the patterns differed between the two ruminant species. Ruminal enzyme activity varied with season, and production of xylanase and cellulase was greater in yak compared to cattle in both fall and winter. The rumen bacterial community varied with season in both yak and cattle, with higher alpha diversity and similarity (beta diversity) in yak than cattle. The diversity indices of eukaryotic community did not change with season in both ruminant species, but higher similarity was observed in yak. In addition, the similarity of rumen microbiome functional community was higher in yak than cattle across seasons. Moreover, yak rumen microbiome encoded more genes (GH2 and GH3) related to cellulose and hemicellulose degradation compared to cattle, and a new enzyme family (GH160) gene involved in oligosaccharides was uniquely detected in yak rumen. The season affected microbiome attenuation and buffering values (stability), with higher buffering value in yak rumen microbiome than cattle. Positive correlations between antimicrobial resistance gene (*dfrF*) and CAZyme family (GH113) and microbiome stability were identified in yak, but such relationship was negatively correlated in cattle.

**Conclusions:**

The findings of the potential of cellulose degradation, the relationship between rumen microbial stability and the abundance of functional genes varied differently across seasons and between yak and cattle provide insight into the mechanisms that may underpin their divergent adaptation patterns to the harsh climate of the Qinghai-Tibetan Plateau. These results lay a solid foundation for developing strategies to maintain and improve rumen microbiome stability and dig out the potential candidates for manufacturing lignocellulolytic enzymes in the yak rumen to enhance ruminants’ performance under extreme environmental conditions.

**Supplementary Information:**

The online version contains supplementary material available at 10.1186/s12915-024-02035-4.

## Background

Microbiome stability is defined as the ability of a microbial community to return to its pre-disturbed state after a disruption, and a greater stability indicates that the community is more resilient to perturbation [1]. A stable gut microbiome is critical for maintaining the host physiology and health [2], and instability is associated with health problems in rats such as esophageal tumor, etc. [3]. In ruminants, a stable rumen microbiome confers the capability to efficiently hydrolyze recalcitrant plant fiber and polysaccharides [4, 5], providing digestible nutrients to the animals for their nutrient requirement. The microbial diversity and the physicochemical properties of the rumen environment (e.g., pH) are fundamental forces driving the stability of the rumen microbiome when it is subjected to perturbation induced by the feed availability and rearing environment [6]. Recently microbial composition has been reported to involve in maintaining the stability of rumen microbial ecosystem in goats [7]. The consonance of the fundamental forces with the rumen microbiome stability has led researchers to unravel the factors that enable the host to utilize feedstuffs more efficiently while emitting less methane [8, 9]. However, the rumen microbiome research has mainly focused on beef and dairy operations under the well managed environments [6–9]. Knowledge of the factors (microbial and host) underpinning rumen microbiome stability in domesticated and non-domesticated grazing animals reared in harsher environments is still lacking. As such, identifying host-microbial mechanisms that impart resilience on the gut microbiomes of these animals may provide clues as to adaptation strategies used by rumen microbes.

The yak raised in extreme environments (extremely cold, low-level oxygen, intense UV radiation, and shortage of nutrients) like the Qinghai-Tibetan Plateau exhibit higher nitrogen utilization efficiency, greater volatile fatty acid production, and less methane production than cattle raised under the same management conditions [10, 11]. Yak harbors a rumen microbiome distinct from that of cattle and sheep even when they are fed on atypical high-grain diet [11]. Although the volume of research concerning the rumen microbial community of grazing ruminants like yak is increasing [12–17], most studies to date only examined the rumen microbiota of animals reared on nutrient-rich grasslands [14, 15], employed 16S rRNA gene sequencing to generate metataxonomic profiles [13, 15–17], and used samples collected at a single or small number of timepoints to reveal the dynamic change patterns of rumen microbiota. To date, most studies on yak rumen research mainly focused on bacteria using amplicon sequencing under feedlot system and little research was conducted to explore the yak rumen microbiome using metagenome [18–20]. Only a few studies focused on the seasonal shift of yak rumen microbiota under grazing conditions with most of them focused on bacteria or fungi using amplicon sequencing with only one study based on metagenome [21–23]. As such, these data do not reveal the integrated compositional and functional profiles of the rumen microbiome, nor do they show seasonal variation and adaptation in rumen microbial stability when animals are grazed on natural pasture. Additionally, the abundance of genes encoded glycoside hydrolases that involved in cellulose and hemicellulose degradation and the activity of carboxymethyl cellulase were much higher in the rumen of yak than cattle under grazing conditions [24], suggesting stronger fiber digestibility in the rumen of yak. Therefore, we speculated that the yak rumen microbiome is more stable than the cattle rumen microbiome due to its higher capacity to digest low-quality forages, and the adaptation of its microbiome to differences brought about by the harsh environment of the Qinghai-Tibetan Plateau. This study employed metagenomic sequencing to compare the rumen microbiome of yak and cattle grazed on the Qinghai-Tibetan Plateau from spring to winter and investigated the dynamic patterns of the rumen microbiome stability throughout the year.

## Results

### Grass chemical composition, rumen fermentation measurement and enzyme activity in yak and cattle across seasons

The chemical composition of grass samples (on a dry matter basis) differed across four seasons (from spring to winter) (Additional file 1: Table S1), with neutral detergent fiber (NDF) and acid detergent fiber (ADF) increasing with season and crude protein (CP, *P* < 0.01) content decreasing with season. The concentration of total VFA (TVFA; mmol/L) in the rumen of grazing yak increased from spring to fall and then decreased in winter, and it was higher in summer and winter in cattle compared to yak (Table 1, *P* < 0.01). The ruminal concentrations of acetate and propionate increased from spring to fall and decreased afterwards in yak, while they reached the highest value in summer and then decreased to remain stable afterwards in cattle (Table 1, *P* < 0.05). Ruminal xylanase and protease activity increased throughout the year although there was a fluctuation in winter (Table 1, *P* < 0.05), and protease activity in fall and winter was numerically higher than that in spring and summer for both ruminant species (Table 1). Amylase activity decreased from spring to winter and cellulase activity increased from spring to fall and then decreased in winter for both yak and cattle (Table 1, *P* < 0.05).

**Table 1:** Rumen fermentation profiles and enzyme activities of grazing yak and cattle at different seasons

Item	Species	Season^1^				SEM	*P*-value		
		Spring	Summer	Fall	Winter		Species	Season	Interaction
pH	Yak	7.47^a^	7.45^aA^	7.23^bB^	7.29^bA^	0.04	0.045	< 0.001	< 0.001
	Cattle	7.88^a^	7.35^bB^	7.46^aA^	7.11^cB^				
Ammonia N(mg/dL)	Yak	16.46^aA^	11.3^bA^	7.12^c^	6.78^cA^	0.65	< 0.001	< 0.001	< 0.001
	Cattle	5.72^aB^	7.31^aB^	6.29^a^	2.13^bB^				
TVFA (mmol/L)	Yak	69.03^a^	68.32^bB^	73.07^aA^	57.52^cB^	1.28	< 0.001	< 0.001	< 0.001
	Cattle	67.43^b^	86.36^aA^	62.96^cB^	68.57^bA^				
Acetate (mmol/L)	Yak	48.66^b^	48.22^bB^	54.57^aA^	42.91^cB^	0.96	< 0.001	< 0.001	< 0.001
	Cattle	48.11^b^	63.75^aA^	48.41^bcB^	52.72^cA^				
Propionate (mmol/L)	Yak	9.18^cB^	10.66^bB^	11.91^aA^	9.29^bc^	0.2	< 0.001	< 0.001	< 0.001
	Cattle	10.9^bA^	13.35^aA^	10.59^bB^	10.81^b^				
Butyrate (mmol/L)	Yak	7.89^aA^	6.69^b^	5.21^cA^	4.05^ dB^	0.21	< 0.001	< 0.001	< 0.001
	Cattle	6.14^aB^	6.46^a^	3.97^cB^	5.05^bA^				
Isobutyrate (mmol/L)	Yak	1.19^a^	1.2^aA^	0.65^bA^	NA^c^	0.08	< 0.001	< 0.001	< 0.001
	Cattle	1.11^a^	1.06^aB^	NA^bB^	NA^b^				
Valerate (mmol/L)	Yak	0.65^aA^	NA^bB^	NA^b^	NA^b^	0.04	< 0.001	< 0.001	< 0.001
	Cattle	NA^bB^	0.57^aA^	NA^b^	NA^b^				
Isovlerate (mmol/L)	Yak	1.47^aA^	1.52^aB^	0.73^bA^	0.63^bA^	0.09	< 0.001	< 0.001	< 0.001
	Cattle	1.17^aB^	1.66^aA^	NA^bB^	NA^bB^				
A:P	Yak	5.3^a^	4.54^b^	4.58^b^	4.32^b^	0.05	0.65	0.003	< 0.001
	Cattle	4.41^b^	4.78^a^	4.59^ab^	4.88^a^				
Xylanase	Yak	1.25^c^	2.27^b^	5.44^a^	5.46^aA^	0.67	< 0.001	0.004	0.15
	Cattle	1.46^b^	1.8^b^	4.86^a^	4.86^aB^				
Protease	Yak	34.02^ab^	29.56^b^	37.03^a^	36.41^ab^	1.03	0.3	0.009	0.51
	Cattle	30.1^ab^	29.56^b^	37.09^a^	34.16^ab^				
Amylase	Yak	32.81^aB^	27.09^b^	22.12^cA^	27.17^bA^	6.08	< 0.001	< 0.001	< 0.001
	Cattle	53.71^aA^	28.19^b^	8.85^bB^	9.42^bB^				
Cellulase	Yak	4.43^bA^	4.3^bA^	13.42^aA^	10.4^aA^	1.59	< 0.001	0.001	< 0.001
	Cattle	0.91^cB^	2.77^aB^	2.77^aB^	1.33^bB^				

^1^Means with different letters in the same row (a–d) or column (A–B) indicate a significant difference, NA means not available. Units of enzyme activity are: Cellulase (umol glucose/min/mL), Amylase (umol glucose/min/mL), Xylanase (umol xylose/min/mL), and Protease (ug hydrolysed protein/h/mL), A:P means the ratio of Acetate/ Propionate

### Overview of yak and cattle rumen metagenomes

**Fig. 1:**
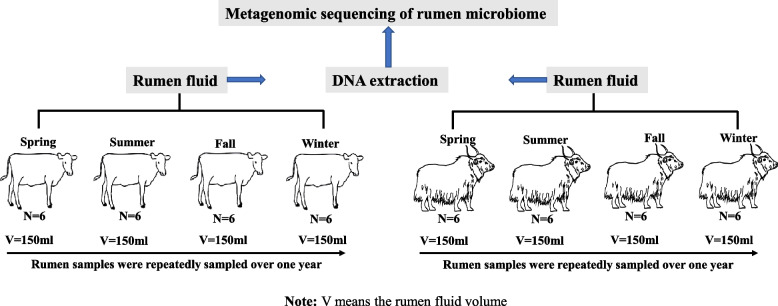
Experimental design for rumen sample collection from grazing yak (*n* = 6) and cattle (*n* = 6) across seasons

**Fig. 2:**
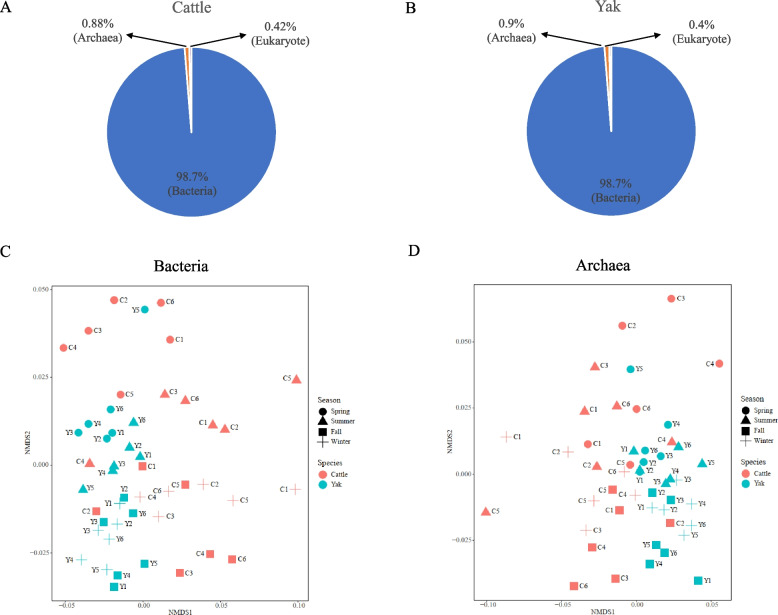
Microbial compositional profiles between yak (*n* = 6) and cattle (*n* = 6) at different seasons. Microbial domains composition between cattle (**A**) and yak (**B**). Non-metric multidimensional scaling (NMDS) analysis plots based on Bray–Curtis metrics showed bacterial (**C**) and archaeal (**D**) communities (at species level) of yak and cattle are different from each other at different seasons

A total of 3,076,178,385 reads, with 64,087,049 ± 994,889 (mean ± SEM) per sample (Additional file 1: Table S2), were obtained from 48 rumen samples collected from grazing yak and cattle throughout the year (Fig. 1). Following quality control and host contamination removal, 60,153,862 ± 822,590 reads were obtained and used for downstream analysis. Assembly of these reads generated 1,335,886 ± 27,741 contigs (N50 of 604 ± 10 bp) (Additional file 1: Table S2). From the classified reads of cattle metagenome, on average 98.7%, 0.88%, and 0.42% were of bacterial, archaeal, and eukaryotic origin (0.22% for fungi and 0.2% for protozoa), respectively (Fig. 2A), while the yak rumen metagenome contained 98.7% of bacterial, 0.9% archaeal, and 0.4% eukaryotic sequences (0.27% for fungi and 0.13% for protozoa), respectively (Fig. 2B). There were significant differences in bacterial and archaeal composition at species level between the two ruminant species (bacteria: ANOSIM *R* = 0.09, *P* = 0.02; archaea: ANOSIM *R* = 0.21, *P* < 0.001) and across seasons (bacteria: ANOSIM *R* = 0.67, *P* < 0.001; archaea: ANOSIM *R* = 0.18, *P* < 0.001) (Fig. 2C, D). Less variation (Bray–Curtis distance) in profiles of bacterial community was evident compared to the archaeal community across seasons in both yak and cattle (Additional file 2: Fig. S1) (Mann–Whitney U test, *P* < 0.001 for Yak; *P* < 0.001 for cattle). No difference was found on the eukaryotic composition across seasons (fungi: ANOSIM *R* = 0.04, *P* = 0.1; protozoa: ANOSIM *R* = 0.06, *P* = 0.06) but they were different between yak and cattle (fungi: ANOSIM *R* = 0.26, *P* = 1e-04; protozoa: ANOSIM *R* = 0.21, *P* = 3e-04, Additional file 2: Fig. S2), and less variation (Bray–Curtis distance) was found in yak compared to cattle for both fungi and protozoa (Mann–Whitney U test, *P* < 0.001, Additional file 2: Fig. S2).

### Diversity and composition of rumen microbiome in yak and cattle

**Fig. 3:**
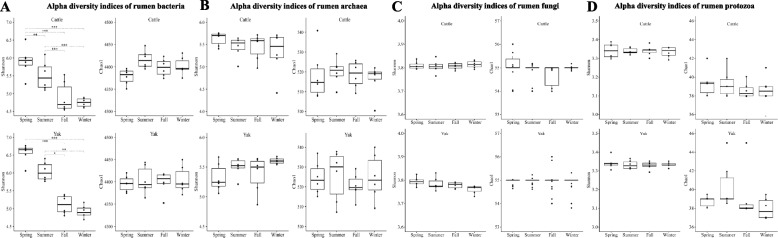
Temporal changes of rumen microbial alpha diversity between yak (*n* = 6) and cattle (*n* = 6) at different seasons. **A** Bacterial Shannon and Chao1 of yak and cattle at different seasons. **B** Archaeal Shannon and Chao1 of yak and cattle at different seasons. **C** Fungal Shannon and Chao1 of yak and cattle at different seasons. **D** Protozoal Shannon and Chao1 of yak and cattle at different seasons. Differences in data were assessed by non-parametric Kruskal–Wallis test in combination with Dunn’s post-doc test for multiple comparisons, and all *P* values were corrected by Benjamin-Hochberg algorithm (* 0.05 < *P* < 0.01, ** 0.01 < *P* < 0.001, *** *P* < 0.001)

The Shannon index of rumen bacterial communities significantly decreased from spring to winter (Fig. 3A, *P* < 0.01), while there was no significant difference in Shannon and Chao1 indices of rumen archaeal communities across seasons in both yak and cattle (Fig. 3B). For fungi and protozoa, there was no significant difference in Shannon and Chao1 indices in both yak and cattle across seasons (Fig. 3C, D). Taxonomic analysis using KRAKEN2 with an internal reference (see details in Methods) identified 38 bacterial phyla, 349 bacterial families, 1207 bacterial genera, and 4513 bacterial species in cattle, accounting for 97.64%, 52.55%, 27%, and 25.12% of the total bacterial sequences, respectively. In addition, 6 archaeal phyla (account for 99.76%), 39 families (account for 91.91%), 135 archaeal genera (account for 89.72%) and 545 archaeal species (account for 76.94%) were identified in cattle. The number of taxa at different taxonomic levels (phylum, family, genus, and species) in the rumen of yak was similar to that found in cattle and bacterial phyla, families, genera and species accounted for 97.64%, 48.34%, 24.76%, and 22.82% of bacterial sequences respectively, and archaeal phyla, families, genera and species accounted for 99.73%, 91.89%, 89.2% and 76.07% of archaeal sequences.

**Fig. 4:**
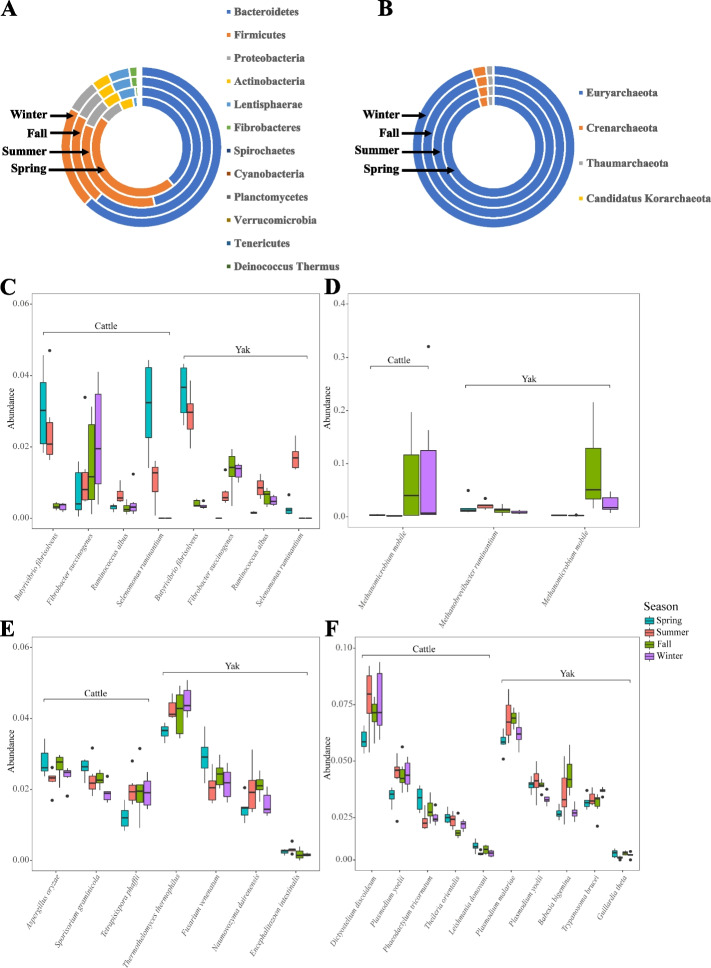
Dynamic change in composition of rumen microbiome between yak (*n* = 6) and cattle (*n* = 6) at different seasons. Composition of rumen bacteria (**A**) and archaea (**B**) at the phylum level (relative abundance > 0.1% in at least in 50% of the at least one timepoint within each species). Significantly different (*P* < 0.05) bacterial (**C**), archaeal (**D**), fungal (**E**), and protozoal (**F**) species between cattle and yak across seasons. Significantly different was assessed by non-parametric Kruskal–Wallis test in combination with Dunn’s post-doc test for multiple comparisons

From identified bacteria taxa, a total of 12 phyla and 67 species were defined as detectable (with relative abundance > 0.1% in at least 50% of animal population in each season; Additional file 1: Table S3 and S4). Among them, phyla Bacteroidetes (cattle vs. yak, 52.76% vs. 52.58%), Firmicutes (30.41% vs. 29.91%), Proteobacteria (6.79% vs. 7.17%), and Actinobacteria (3.78% vs. 4.27%) were dominant in the rumen of both ruminant species (Fig. 4A, Additional file 1: Table S3). At the genus level, the dominant genera were *Prevotella* (cattle vs. yak, 32.78% vs. 28.17%), *Butyrivibrio* (4.5% vs. 5.55%), *Ruminococcus* (4.35% vs. 2.96%), *Fibrobacter* (4.2% vs. 3.46%), *Streptomyces* (3.17% vs. 3.86%). The predominant species consisted of *Butyrivibrio proteoclasticus* (1.84% vs. 2.5%), *Butyrivibrio fibrisolvens* (1.56% vs. 1.81%), *Butyrivibrio hungatei* (1.35% vs. 1.63%), *Fibrobacter succinogenes* (1.35% vs. 0.84%), and *Prevotella ruminicola* (1.13% vs. 0.92%) in the rumen of both cattle and yak (Additional file 1: Table S4). For archaea, a total of 4 phyla and 364 species were detectable (with relative abundance > 0.1% in at least 50% of animal population in each season, Additional file 1: Table S5 and S6). Of these, the predominant phylum was Euryarchaeota (94.93% vs. 96.13%), followed by Crenarchaeota (2.99% vs. 2.43%), Thaumarchaeota (1.85% vs. 1.27%), and Candidatus Korarchaeota (0.14% vs. 0.06%) in the rumen of both ruminant species (Fig. 4B, Additional file 1: Table S5). The predominant archaeal genus was *Methanobrevibacter* (14.94% vs. 15.82%), followed by *Halorubrum* (8.6% vs. 9.37%), *Methanosarcina* (3.48% vs. 3.1%), and *Methanomicrobium* (3.48% vs. 2.51%). The dominant species were *Methanomicrobium mobile* (3.97% vs. 2.9%), *Methanobrevibacter olleyae* (0.98% vs. 1.67%) and *Methanobrevibacter ruminantium* (0.98% vs. 1.53%) in both cattle and yak (Additional file 1: Table S6).

For the fungal community, 3 phyla and 41 species were identified with phyla Ascomycota (cattle vs. yak, 90.49% vs. 90.01%) and Basidiomycota (8.68% vs. 9.18%) being dominant in both yak and cattle. At the genus level, the dominant genera were *Fusarium* (15.07% vs. 15.03%), *Pyricularia* (6.97% vs. 6.9%), *Colletotrichum* (4.76% vs. 6.13%), and *Candida* (5.67% vs. 5.07%). The predominant fungal species were *Colletotrichum higginsianum* (4.89% vs. 6.29%), *Thielavia, terrestris* (4.47% vs. 4.97%), *Botrytis cinerea* (4.16% vs. 3.93%), and *Thermothelomyces thermophilus* (3.54% vs. 4.14%). For protozoa, a total of 7 phyla and 39 species were detected, and 99.2% protozoal sequences were assigned to phyla Apicomplexa (71.08% vs. 69.07%), Kinetoplastida (17.22% vs. 19.87%), Dictyosteliales (5.97% vs. 5.06%), and Bacillariophyta (4.59% vs. 5.22%). At the genus level, genera *Plasmodium* (44.06% vs. 40.1%), *Leishmania* (14.43% vs. 17.41%), *Besnoitia* (6.09% vs. 7.13%), and *Toxoplasma* (5.34% vs. 6.01%) were dominant in both yak and cattle. At the species level, the predominant species were *Besnoitia besnoiti* (6.98% vs. 8.2%), *Plasmodium malariae* (6.91% vs. 6.2%), *Toxoplasma gondii* (6.11% vs. 6.9%), and *Dictyostelium discoideum* (6.85% vs. 5.85%).

### Compositional changes of rumen microbiome in yak and cattle through four seasons

Further differential abundance comparison at the phylum level revealed that the relative abundance of 5 bacterial phyla (Bacteroidetes, Verrucomicrobia, Tenericutes, Firmicutes, and Actinobacteria) in cattle were different across seasons (Additional file 2: Fig. S3, *P* < 0.05). In yak, the relative abundance of 11 phyla (Bacteroidetes, Firmicutes, Proteobacteria, Actinobacteria, Lentisphaerae, Fibrobacteres, Spirochaetes, Cyanobacteria, Planctomycetes, Verrucomicrobia, and Deinococcus-Thermus) showed seasonal changes (Additional file 2: Fig. S3, *P* < 0.05). At species level, the relative abundances of 6 taxa involved in different fermentation functions showed seasonal change in both yak and cattle. Particularly, the relative abundance of *Fibrobacter succinogenes* (fiber degrader) increased from spring to winter, whereas *Selenomonas ruminantium* (sugar fermenter) decreased across seasons (Fig. 4C). For archaea, the relative abundance of *Methanomicrobium mobile* significantly increased from summer to winter in both yak and cattle, while the relative abundance of *Methanobrevibacter ruminantium* significantly decreased from summer to winter in yak (Fig. 4D).

For fungi, the relative abundances of *Aspergillus oryzae*, *Sporisorium graminicola*, and *Tetrapisispora phaffii* varied significantly with season in cattle, while those of *Thermothelomyces thermophilus*, *Fusarium venenatum*, *Naumovozyma dairenensis*, and *Encephalitozoon intestinalis* fluctuated greatly across seasons in yak (Fig. 4E). Regarding to protozoa, the relative abundances of five species including *Dictyostelium discoideum*, *Plasmodium yoelii*, *Phaeodactylum tricornutum*, *Theileria orientalis*, and *Leishmania donovani* varied significantly across seasons in cattle, while that of *Plasmodium malariae*, *Plasmodium yoelii*, *Babesia bigemina*, *Trypanosoma brucei*, and *Guillardia theta* showed significantly different across seasons in yak (Fig. 4F).

### Dynamic functional changes of the rumen microbiome in yak and cattle through the year grazing

**Fig. 5:**
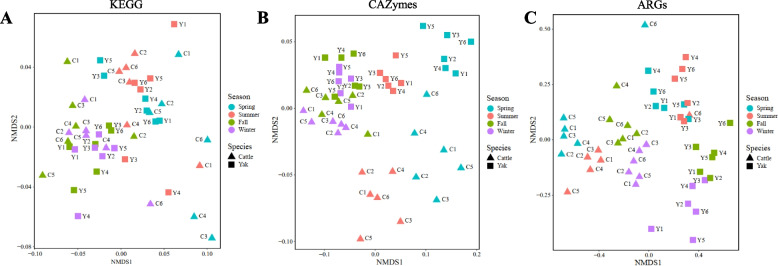
Temporal changes of rumen functional profiles between yak (*n* = 6) and cattle (*n* = 6) at different seasons. Non-metric multidimensional scaling (NMDS) analysis plots based on Bray–Curtis metrics showed KEGG profiles (**A**, at level 2), CAZyme profiles (**B**), and ARGs profiles (**C**) between yak and cattle at different seasons

**Fig. 6:**
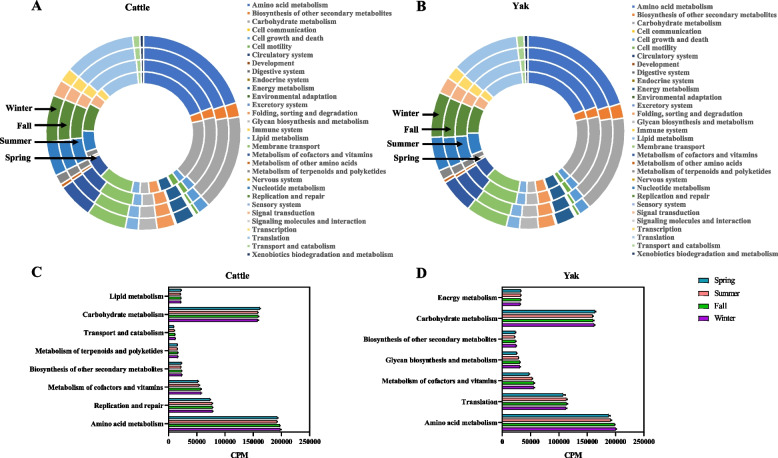
Rumen microbiome KEGG pathways function between yak (*n* = 6) and cattle (*n* = 6) at different seasons. Composition of KEGG (level 2) at different seasons between cattle (**A**) and yak (**B**). Significantly different (*P* < 0.05) KEGG pathways between cattle (**C**) and yak (**D**) across seasons. Significantly different was assessed by non-parametric Kruskal–Wallis test in combination with Dunn’s post-doc test for multiple comparisons

Functional analysis showed that both host species and season affected the functional profiles of the rumen microbiome (Fig. 5A-C). A total of 31 level 2 KEGG pathways were identified with “Amino acid metabolism”, “Carbohydrate metabolism”, “Translation”, “Membrane transport”, “Replication and repair” being predominant in both cattle and yak (Fig. 6A, B, Additional file 1: Table S7). The abundance (counts per million, CPM) of five KEGG pathways (Biosynthesis of other secondary metabolites, Glycan biosynthesis and metabolism, Metabolism of cofactors and vitamins, Translation, and Amino acid metabolism) in yak rumen microbiome significantly increased throughout the year, while six of them increased in cattle rumen microbiome including Transport and catabolism, Metabolism of terpenoids and polyketides, Biosynthesis of other secondary metabolites, Metabolism of cofactors and vitamins, Replication and repair, and Amino acid metabolism (Fig. 6C, D, *P* < 0.05). The abundance of “Carbohydrate metabolism” significantly decreased from spring to summer and then increased afterwards (Fig. 6C, D, *P* < 0.05), while those of “Energy metabolism” and “Lipid metabolism” decreased through the year for both ruminant species (Fig. 6C, D, *P* < 0.05). Among the functions identified, this study focused on CAZymes and ARGs below due to the former provides many carbohydrate-active enzymes to digest forage polysaccharides efficiently and the latter contributes to microbial success and survival within complex ecological systems, which may affect the rumen microbiome stability.

### Differences in the seasonal changes of CAZyme profiles between yak and cattle

**Fig. 7:**
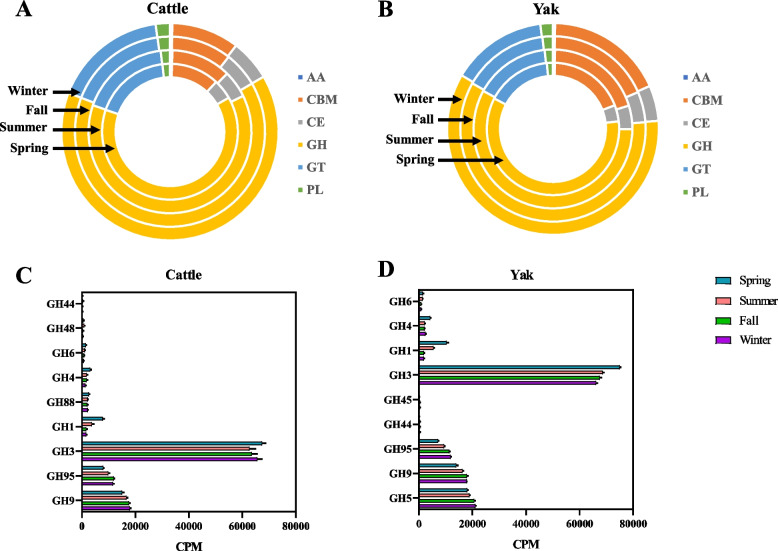
Rumen microbiome CAZymes between yak (*n* = 6) and cattle (*n* = 6) at different seasons. Composition of CAZymes at different seasons between cattle (**A**) and yak (**B**). Significantly different (*P* < 0.05) CAZyme families between cattle (**C**) and yak (**D**) across seasons. Significantly different was assessed by non-parametric Kruskal–Wallis test in combination with Dunn’s post-doc test for multiple comparisons

CAZyme genes including those encoding glycoside hydrolases (GHs), glycosyl transferases (GTs), carbohydrate esterases (CEs), carbohydrate-binding modules (CBMs), polysaccharide lyases (PLs), and auxiliary activities (AAs) were identified from rumen microbiome and are presented in Table S8 and Fig. 7A, B. Comparison of CAZyme gene families showed that the numbers of CAZyme genes in the rumen microbiome was similar between yak (402) and cattle (397), but 18 of them were unique in cattle and 23 of them were only detected in yak (Additional file 2: Fig. S4A). Additionally, the CAZyme gene profiles were also influenced by season with several CAZyme gene families being uniquely associated with the season for each ruminant species (Additional file 2: Fig. S4B). For example, AA0 (spring), CBM19 (summer), GH13_6 (fall) and GH91 (winter) genes were only detected in cattle, GH13_1 (spring), GT82 (summer), GH13_35 (fall) and GH160 (winter) genes were only detected in yak. The abundance of genes for GHs associated with cellulose, hemicellulose, and starch degradation was also evaluated and some of them only detected in a particular season and/or in either ruminant species. Specifically, GH3 was the predominant CAZyme family throughout the year in the rumen of both ruminant species (Additional file 1: Table. S8) and several GH families involved in cellulose degradation varied significantly with the season in both yak and cattle (Fig. 7 C, D, *P*  < 0.05). For example, the abundance of GH95 and GH9 in cattle increased from spring to fall (Fig. 7C), and that of GH95, GH9 and GH5 in yak increased from spring to winter (Fig. 7D).

To identify the microbial provenance of CAZyme genes, the contigs containing CAZyme sequences were assigned to rumen metagenome-assembled genomes (MAGs) using Kraken2. Twenty bacterial species were identified to contain CAZyme-encoding genes, with *F. succinogenes* and *R. flavefaciens* harboring the highest number of CAZyme genes (167 and 144, respectively) followed by *S. ruminantium* (87), *Eubacterium ruminantium* (71), and *Prevotella bryantii* (67) (Additional file 1: Table. S9).

### Seasonal change of antimicrobial resistance gene (ARG) profiles in the rumen microbiome of yak and cattle

**Fig. 8:**
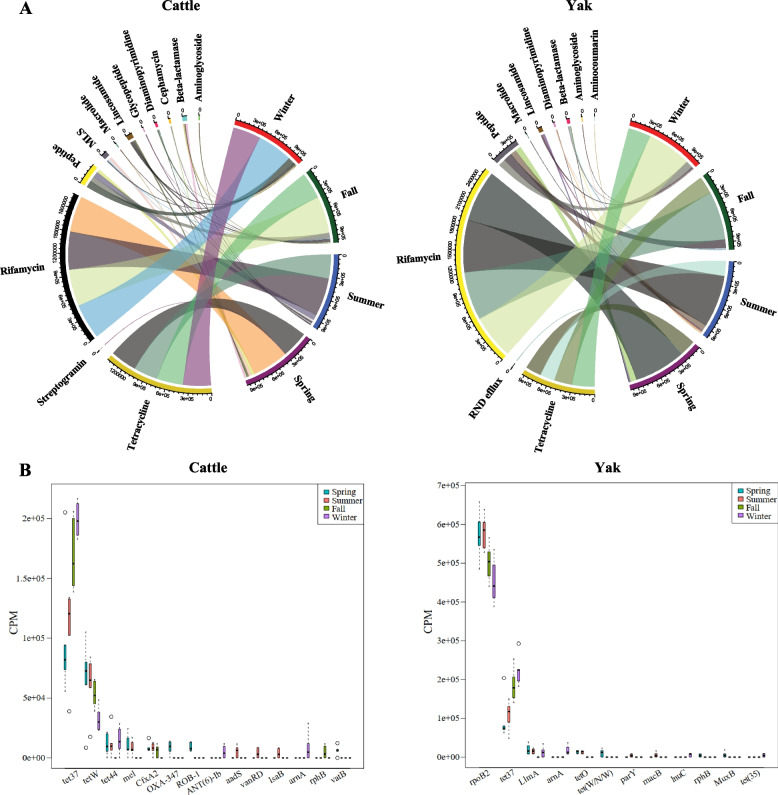
Rumen microbiome ARGs profile between yak (*n* = 6) and cattle (*n* = 6) at different seasons. Composition of ARGs at different seasons between cattle (**A**) and yak (**B**). Significantly different (*P* < 0.05) ARGs between cattle (**C**) and yak (**D**) across seasons. Significant difference was assessed by non-parametric Kruskal–Wallis test in combination with Dunn’s post-doc test for multiple comparisons, and the circos plots were generated using circlize R package [128]

In total, 34 ARGs were detected in the rumen microbiome and grouped into 14 antibiotic types: rifamycin, tetracycline, peptide, lincosamide, and beta-lactamase being the most abundant in both cattle and yak (Fig. 8A, B, Additional file 1: Table. S10). Of these, the relative abundance of 14 ARGs shifted seasonally in the rumen of cattle (Fig. 8C), while that of 12 ARGs varied across seasons in the rumen of yak (Fig. 8D). *Tet37*, *tetW*, *tetQ*, *ugd*, *rpoB2*, and *rpoB* were ubiquitous across all rumen samples, four (*parY*, *AAC(6’)-Ie-APH(2’’)-Ia*, *MuxB* and *tet(35)*) were detected exclusively in yak rumen microbiome, while thirteen known ARGs (*ANT(6)-Ib*, *aadS*, *OXA-347*, *ROB-1*, *CfxA2*, *vanRG*, *vanRD*, *ErmF*, *mel*, *lsaB*, *vatB*, *tet44* and *tetX*) were only found in cattle rumen microbiome (Additional file 1: Table. S10). From 22 of significantly different ARGs between yak and cattle (LDA score > 2 and *P* < 0.05), 17 of them (*tetO*, *tetX*, *aadS*, *ErmF*, *vatB*, *CfxA2*, *ANT(6)-Ib*, *mel*, *vanRG*, *lnuC*, *tet44*, *tetW*, *ROB-1*, *OXA-347*, *tet(W/N/W)*, *tet(40)*, and *tetQ*) had higher abundance in cattle and 5 (*ugd*, *arnA*, *rpoB*, *rpoB2*, and *AAC(6')-Ie-APH(2'')-Ia*) showed higher abundance in yak (Additional file 1: Table. S10).

The ARG-containing contigs were assigned to rumen MAGs and showed that *E. ruminantium*, *F. succinogenes*, *R. flavefaciens*, and *S. ruminantium* conferred the majority of ARGs (Additional file 1: Table. S11). Notably, eleven bacteria were found to carry both CAZyme and ARG-genes with five (*E. ruminantium*, *F. succinogenes*, *R. flavefaciens*, *S. ruminantium*, and *Sharpea azabuensis*) of them shared by yak and cattle and three species were detected uniquely in the rumen of each ruminant species (*Bacillus licheniformis*, *Eubacterium pyruvativorans*, and *Lachnobacterium bovis* in yak; *Erysipelothrix rhusiopathiae*, *Ruminococcus bromii*, and *Selenomonas bovis* in cattle) (Additional file 2: Fig. S5A). Of these, *R. flavefaciens* and *S. ruminantium* were ubiquitous in the rumen of both yak and cattle across all seasons (Additional file 2: Fig. S5B), however, the profile of CAZymes and ARGs genes they carry was divergent between yak and cattle (Additional file 1: Table. S12).

### Seasonal rumen microbiome stability and its relationship with microbial functions

**Fig. 9:**
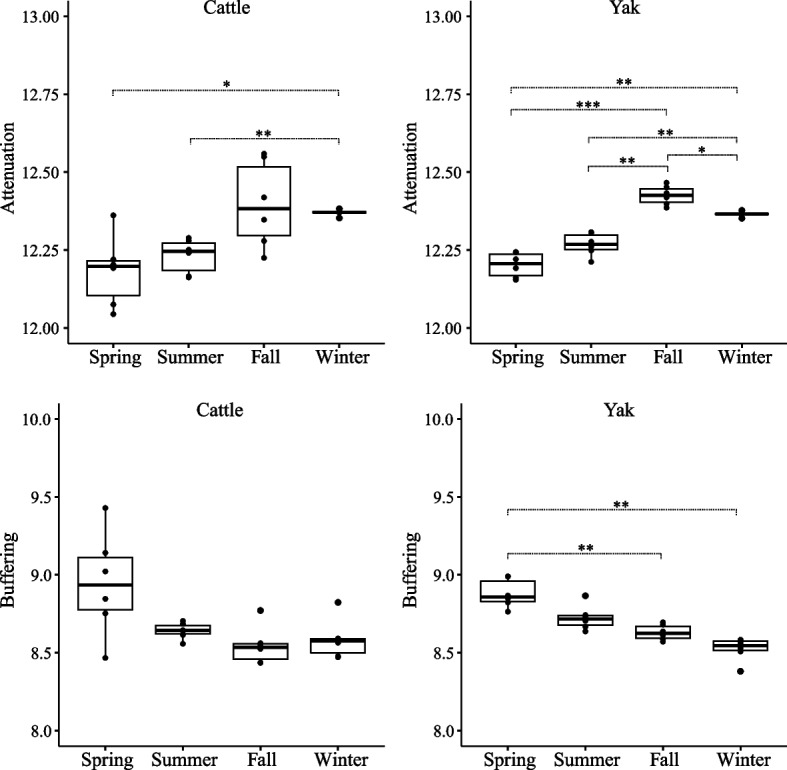
Temporal changes of attenuation and buffering values in rumen microbiota between yak (*n* = 6) and cattle (*n* = 6) across seasons. Statistical analysis was determined using repeated measures ANOVA and the Tukey test for multiple comparisons, and *P* values were corrected by Benjamin-Hochberg algorithm (* 0.05 < *P* < 0.01, ** 0.01 < *P* < 0.001, *** *P* < 0.001)

The temporal stability of rumen microbiome in yak and cattle across seasons was assessed by calculating attenuation and buffering indices (See methods section for details). The attenuation value of the rumen microbiome in yak and cattle significantly increased from spring to fall and then decreased afterwards (Fig. 9,* P * < 0.05). The buffering value of yak rumen microbiome significantly decreased across seasons (Fig. 9,* P* < 0.01), while that of cattle was not statistically significant (Fig. 9).

**Fig. 10:**
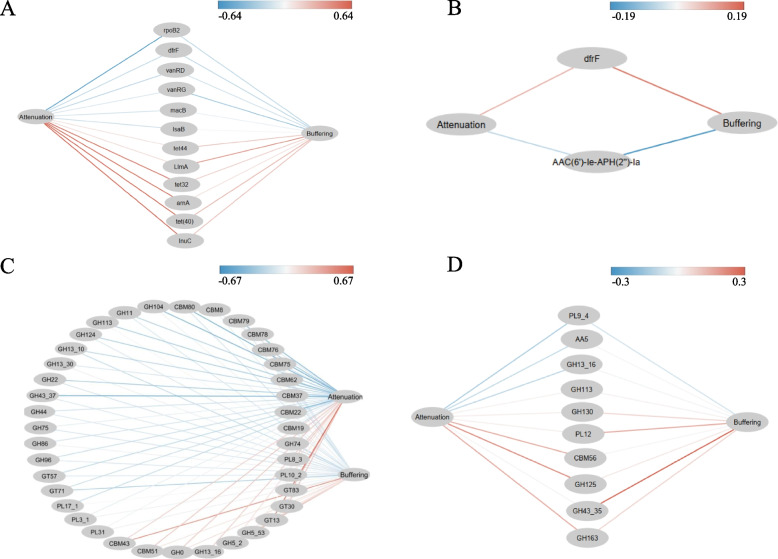
The potential relationship among ARGs, CAZymes and stability (Attenuation and Buffering). Correlation analysis was performed by Spearman rank correlation, and only correlations (among ARGs, CAZymes and stability) that were positive or negative at the same time were displayed. **A** Relationship between ARGs and stability in cattle. **B** Relationship between ARGs and stability in yak. **C** Relationship between CAZymes and stability in cattle. **D** Relationship between CAZymes and stability in yak. The lines in red and blue denote positive and negative correlations, respectively

Both positive or negative relationships between ARGs/CAZYmes genes and attenuation and buffering indices were identified. In the rumen of cattle, the abundance of 6 ARGs genes (*arnA*, *lnuC* (*F. succinogenes*), *tet32*, *LlmA* (*R. bromii*), *tet (40)* and *tet44*)) were positively correlated with the microbiome stability (0.08 < *R* < 0.64, 0.001 < *P* < 0.68), while the abundance of *dfrF*, *rpoB2* (*F. succinogenes, R. flavefaciens*, and *S. ruminantium*), *vanRD*, *vanRG* (*E. ruminantium*), and *lsaB* (*R. flavefaciens*) had negative relationship with stability measures (-0.57 < *R* < -0.0076, 0.003 < *P* < 0.97, Fig. 10A). In the rumen of yak, only *dfrF* (*E. ruminantium*) had positive correlation with the microbiome stability (0.08 < *R* < 0.17, 0.42 < *P* < 0.84), while that of *AAC (6’)-Ie-APH (2’’)-Ia* negatively correlated with microbiome stability (-0.19 < *R* < -0.07, 0.36 < *P* < 0.78, Fig. 10B). For CAZymes, the abundance of majority of GH and CBM families had negative correlations with stability in the cattle (-0.56 *R* < -0.001, 0.004 < *P* < 0.94, Fig. 10C), whereas most of them had positive correlations in the rumen of yak (0.03 < *R* < 0.30, 0.15 < *P* < 0.95, Fig. 10D). Interestingly, positive relationship among GH113 (*E. ruminantium* and *F. succinogenes*), *dfrF* and microbiome stability was found in yak but a negative relationship among the same features in cattle.

## Discussion

The diversity and stability of the rumen microbiome are important to host nutrition, immunity, and health [25]. The higher alpha diversity (Shannon; Mann–Whitney U test, *P* < 0.05) in bacteria and microbial community similarity (beta-diversity; bacteria, archaea, fungi, and protozoa; Mann–Whitney U test, *P* < 0.05) in the rumen of yak across seasons compared to domestic cattle are potential adaptive mechanisms of the yak rumen microbiome to an inhospitable environment. Understanding the microbial adaptation mechanisms in the rumen are vital to understand how ruminants and their symbiotic gut microbes respond to unfavourable external environments.

In this study, the alpha diversity (Shannon) of rumen bacteria in yak was greater than that of cattle across seasons (fiber content increased with season). Generally, higher bacterial diversity improves microbial stability [26], rate of dietary fiber fermentation, and indicates better health status of host and stronger metabolic capacity [27], as well as helps the host to adapt to the high-altitude environments [28]. Indeed, higher stability (buffering value) was observed for the yak rumen microbiome than that of cattle. It was reported that higher microbial stability facilitates the utilization of limited available nutrients [29] and associates with greater performance when diet shifts [30]. Therefore, high rumen microbiome stability in yak could be beneficial to its better adaptation to the dietary changes under harsh environment. Besides, the rumen bacterial and protozoal similarity across seasons in yak was higher than cattle, which enhances its resilience to dietary changes and adaptation to the harsh environment as high similar microorganisms could reinforce their competitiveness under ambient stress [31]. Notably, Rumen protozoa plays an important in plant materials degradation for microbial usage and predates bacteria and fungi, as well as provides methanogens with H_2_ for methane emission [4, 32]. Moreover, it has been demonstrated that protozoa populations shaped the rumen bacteria and archaea community and function [33]. We, therefore, speculate that higher similarity of rumen protozoal community across seasons in yak may be one of the key points making them better adapt to the harsh environment and/or season.

Metagenomic analysis revealed that both of grazing yak and cattle rumen bacteriomes were similar to those previously reported in grazing yak [12, 34], cattle [35, 36], and sheep [37]. Although bacterial composition in the rumen was similar in yak and cattle, the yak microbiome exhibited less seasonal variation than that of cattle as reflected by their lower coefficient of variation (yak vs. cattle, 3.76 vs. 3.80; Mann–Whitney U test, *P* < 0.05). Previous studies showed that the relative abundances of fibrolytic rumen microorganisms increased with dietary fibre content in undomesticated ruminants such as roe deer and yak [38, 39], which is consistent with the data in this study, where the relative abundance of *F. succinogenes* increased with season (increased fiber content). In addition, the increased relative abundance of *Thermothelomyces thermophilus* with season in the rumen of yak, suggesting that this fungal species could facilitate the utilization of fiber as it has been reported to have the capability to produce extracellular lignocellulolytic enzymes [40]. Additionally, our data indicated that cellulase activity in the yak rumen was about fourfold higher than cattle, supporting a previous observation of higher fiber digestibility (NDF, hemicellulose, and ADF) in yak versus cattle [41]. All these suggest that the rumen microbiome of yak has stronger ability to metabolize the fibrous material compared to cattle [24], contributing to its low-quality forage adaptation under hard environment.

We speculated that the functional stability of rumen microbiome across seasons is influenced by both composition and function of its members. Our data showed the dynamic nature of rumen microbial function throughout the year, and its variation according to diet and external environment. Dietary CP content significantly decreased from spring to winter but the abundance of microbial amino acid metabolism-related functions in the rumen significantly increased from spring to winter in both yak and cattle. This indicates that the rumen microbes during fall and winter may be more active to overcome the deficiency of CP, as evidenced by numerically higher protease activity in fall and winter compared to that in spring and summer, which supports previous observations of elevated microbial amino acid metabolism in sheep supplemented with a low amount of urea compared to those fed at a higher rate [37]. Carbohydrate metabolism pathways were enriched in the rumen of both yak and cattle during winter compared to summer. Dietary carbohydrates are metabolized by the rumen microbes to produce lactate and volatile fatty acids (VFAs) via the glycolytic and pentose phosphate pathways [42], the primary energy source for ruminants. Thus, the increases in gene abundance involved in carbohydrate metabolism pathways in the rumen microbiome during winter may be required to maintain the animal normal physiological state to face the reduced feed quality and harsher external conditions.

Carbohydrate metabolism is the principal energy-producing pathway for ruminants and CAZymes are key players in this process [43]. These specialized enzymes degrade the complex plant cell-wall materials and provide the substrates for the growth of various microbes to produce VFAs [44]. We speculated that rumen microbiome stability affects the production of CAZymes, which may, in turn, influence the microbial community in response to a disturbance/perturbation such as changes in dietary fiber intake. Therefore, the changes in the abundance of CAZyme producers (the microbes themselves) and the activity of the CAZymes could contribute to differences in microbiome composition and functional stability between yak and cattle. In the current study, 31 CAZyme families were found to be significantly different in abundance between yak and cattle (LDA score > 2, *P* < 0.05) and more carbohydrate and energy metabolisms functional pathways were enriched in yak than in cattle (LDA score > 2, *P* < 0.05), suggesting that the rumen microbiome of yak may harvest energy from natural pasture more effectively than that of cattle. Among them, the abundance of GH3 and GH2 coding genes, two GH families are associated with the breakdown of plant structural polysaccharides (cellulose and hemicellulose) [45, 46], was 1.1-fold higher in the rumen of yak than those in cattle, indicating that yak had higher cellulase and xylanase activities. The CAZyme families unique to cattle in the current study were previously detected in beef cattle and dairy cows [8, 43, 47–49], suggesting that these CAZyme families are core enzymes across different cattle species. Additionally, host species-specific CAZyme families were identified. For example, GH152 and GH160 were unique to the yak rumen microbiome as observed in the current study and are novel CAZyme families that have not been found in previous yak rumen studies [50–53]. GH152 hydrolyses glucans [54] and GH160 cleaves exopolysaccharides [55], suggesting that the yak rumen microbiome is a rich reservoir of polysaccharide digestive enzymes, and the novel CAZymes gene found in the rumen of yak may be the good candidates for manufacturing enzyme products. Future studies to enrich and express these novel enzymes using the targeted enzyme discovery approach as reported in our previous study [56] will accelerate the implications of these enzymes to improve the fiber degradation in ruminants.

CAZyme abundance profiles changed seasonally in both yak and cattle. The abundance of families involved in cellulose degradation (GH9 and GH95) increased from spring to winter in both ruminant species, corresponding to the increasing fiber content of the grassland. A previous study showed the abundance of GH9 increased linearly with increasing dietary forage content in the rumen of dairy cows [57] and decreased with increasing dietary degradable starch (and decreased fiber content) in the rumen of dairy goats [58], supporting our observations. The majority of GH9 family genes originated from *F. succinogenes* in both yak and cattle, the relative abundance of which also increased with season. This underscores that variation in microbiome composition and function is dependent on substrate availability. Trends of seasonal variation were also observed for GH3, an enzyme involved in glucose production which functions as a rate-limiting enzyme during cellulose degradation [57]. Its abundance increased from summer to winter in cattle, while it decreased from spring to winter in yak. The members of this family possess a variety of enzymatic capabilities, comprising β-D-glucosidase (EC 3.2.1.21), β-D-xylosidase (EC 3.2.1.37), α-Larabinofuranosidase (EC 3.2.1.55), and N-acetyl-β-D-glucosaminidase (EC 3.2.1.52) [59]. Hence the divergence in expression profiles between ruminant species may point to different roles for this family of enzymes in the rumen of yak and cattle. Consistent with this study, the abundance of GH3 decreased from spring to winter in the rumen of Shenzha yak [22]. However, the abundance of GH3 decreased from spring to autumn and then increased afterwards in the rumen of the other three yak breeds [22]. These results suggest that host (species or genetic background) may affect the rumen microbiome [60] and CAZymes profiles [22].

In contrast to earlier studies which found *Prevotella* and *Bacteroides* as the main sources of predicted CAZyme genes in the rumen of Holstein cows and dairy goats [57, 58], *F. succinogenes* and *R. flavefaciens* contributed the most CAZyme genes in both yak and cattle in this study. *Bacteroides*, *Ruminococcus*, and *Fibrobacter* are the dominant fibrolytic microorganisms in the rumen [57], while *Prevotella* species can degrade starch and other plant cell wall polysaccharides [61]. These discrepancies suggest that the profiles of CAZymes producers in the rumen are affected by diet/feed sources and/or host species. Notably, the CAZymes encoded by *F. succinogenes* and *R. flavefaciens* differed between ruminant species, with higher number of GHs genes deriving from both bacteria in yak than in cattle (yak vs. cattle; *F. succinogenes*, 76 vs. 54, *R. flavefaciens*, 64 vs. 43). This further reveals different bacterial adaptation strategies in the rumen of cattle and yak, and that the yak rumen microbiome may exhibit greater capacity for glycoside hydrolase production, potentially contributing to its higher cellulose digestibility versus cattle.

Another microbial function that can affect microbiome stability is the presence of antimicrobial resistance genes (ARGs) by the rumen microbes, which contributes to microbial success and survival within complex ecological systems [62]. Although the yak and cattle were not fed any antibiotics, multiple ARGs were identified within the rumen metagenome (14 classes and 34 individual ARGs), which is consistent with the previous studies cattle raised without antibiotics [61, 63]. The diverse array of ARGs in antimicrobial-free animals may be attributed to the presence of commensal microbiota that naturally carry ARGs. Environmental sources of ARGs, such as the microbiota of soil and water [64, 65], may also contribute to their presence in the rumen of grazing animals. In the current study, the most prevalent ARGs were genes resistant to rifamycin, followed by tetracycline, and peptide, which are inconsistent with those previously reported in the rumen of concentrate- and forage-fed beef cattle, where genes resistant to chloramphenicol were predominant, followed by macrolide and β-lactamase [61]. Additionally, tetracycline, glycopeptide and fluoroquinolone resistant genes were dominant in the rumen of dairy cattle [66]. Moreover, tetracyclines, macrolides, and lincosamides were the most representative antimicrobial resistance gene families in the rumen of Spanish dairy cattle [67]. Diet and host are the key factors that affect the composition of rumen microbiome [4], and the rumen microbiome are the host of ARG genes, thereby divergent in ARG patterns among ruminant species (differences in diet and host) could be expected.

Of the 22 significantly differentially abundant ARGs between yak and cattle, *tetW* was the dominant tetracycline-resistance genes and was more prevalent in the grazing cattle. It has been reported that *tetW* is the predominant gene among annotated ruminal microbial genomes [68] and commonly expressed in the rumen of feedlot cattle [63]. Although tetracycline was not administrated to the cattle in our study and it is unclear where these cattle may have obtained this gene prior to the trial, the ubiquitous detection of *tetW* in cattle suggests that its predominance may be a result of the domestication. The *ANT(6)-Ib* and *tet44* genes were also unique to cattle, and have been detected in the fecal microbiomes of cattle [69] and dairy calves [70], suggesting these two ARG genes are common in the gut of cattle raised in various environments. *ANT(6)-Ib* is an aminoglycoside nucleotidyltransferase gene and confers resistance to streptomycin, while *tet44* encodes ribosomal protection proteins to resistance to tetracycline and minocycline. Both genes originate from *Campylobacter* [71], and members of *Campylobacter* (*Campylobacter fetus subsp. Fetus* and *Campylobacter fetus subsp. venerealis*) inhabit gastrointestinal or reproductive tracts of ruminants including cattle and sheep [72] are food-borne pathogens [73]. Therefore, the prevalence of the antimicrobial resistance gene from the *Campylobacter* strains in the rumen of cattle represents a public health and food safety threat due to potential transmission to humans through food products, including meat and milk [73].

On the other hand, the *rpoB* gene was highly abundant in the rumen of yak. This gene is a target for rifampicin, lipiarmycin, and streptolydigin, and also encodes the β-subunit of RNA polymerase [74]. RNA polymerase (RNAP), a crucial transcription enzyme in all living organisms [75], allows a cell within an organism to adapt to a given environment by regulation of gene expression, thereby maintaining the basic metabolic processes necessary for host survival [76]. Additionally, *AAC(6’)-Ie-APH(2’’)-Ia* was unique to yak. *AAC(6’)-Ie-APH(2’’)-Ia* is an aminoglycoside acetyltransferase encoded by plasmids and transposons in Gram-positive bacteria such as *Enterococcus* [77]. It is involved in phosphorylation and acetylation of various substrates, and bacteria with this gene would have a selective advantage on available substrates under a clinical environment [73]. Therefore, we hypothesized that the higher abundance of *rpoB* and the presence of unique ARGs (*AAC(6’)-Ie-APH(2’’)-Ia*) in the yak rumen may improve rumen microbiome stability under extreme environment, which warrants future research. As ARG profiles have not been reported from rumen microbiome of yak previously, larger sample sizes and the use of animals from different environments may be required in future studies to verify these findings. In addition, the abundance of *rphB* showed opposite trends in yak (decreased from spring to winter) and cattle (increased from spring to fall), suggesting the difference in ARG profiles and their shifts could be driven by host derived rumen microbiome features [78].

Another novel aspect of the current study is that we identified relationships among stability parameters (attenuation and buffering) of microbiome, microbial ARGs, and CAZymes, supporting our hypothesis that these two microbial functions contribute to rumen microbiome stability. In cattle, the *lnuC* and *LlmA* ARGs were positively correlated with stability, while *lsaB*, *vanRG* and *rpoB2* were negatively correlated with stability. *lnuC* is a transposon-mediated nucleotidyltransferase and confers resistance to lincosamide [79], and *LlmA* is 23S ribosomal RNA methyltransferase and confers resistance to lincosamide [80]. Lincosamide was also detected in the gut of beef and dairy cattle [66, 69, 81]. This suggests that lincosamide may play an important role in microbial survival under harsh environments in the rumen of cattle and contributes to improved microbial stability. Notably, host-specific relationships between ARGs and microbiome stability were observed, as evidenced by opposite relationship between *dfrF* and stability in yak (positive) and cattle (negative). *DfrF* is a chromosome-encoded dihydrofolate reductase and confers resistance to trimethoprim [82]. Trimethoprim is present in lettuce and carrot [83], and the natural grass contains many types of antibiotics [84], therefore, natural grass digested by yak may contribute to the appearance of *dfrF*. Moreover, the relationship between stability and CAZyme families were investigated, and host-specific relationships were observed. For example, GH113 was positively correlated with stability in yak and negatively correlated with stability in cattle, and GH13_16 was negatively corelated with stability in yak but had a positive relationship with stability in cattle, suggesting that the relationship between microbiome stability and specific features of the microbiome may be host-specific.

In addition, *R. flavefaciens* and *F. succinogenes* conferred the majority of ARGs in the rumen of both yak and cattle. *R. flavefaciens* and *F. succinogenes* are important CAZyme producers in the rumen [48]. This is consistent with a recent study of the industrialized human fecal microbiome, where many bacterial species have multiple roles including the production of ARG and CAZyme genes [85]. Notably, *F. succinogenes* produced the *lnuC* and GH0 genes that were positively correlated with stability (attenuation and buffering) in cattle, and *R. flavefaciens and F. succinogenes* expressed genes encoding GH125 and GH130 that were positively associated with stability in yak, indicating that these two species may play an important role in maintaining rumen microbiome stability. Previous studies revealed that bacteria could gain novel functions that enable rapid adaptation to a changing environment via horizontal gene transfer, especially when they are under strong selective pressures including from habitats [85], antibiotics and diet [86]. Therefore, we speculated that the CAZymes and ARGs encoded by these two species play an important role in their survival and adaptation of grazing ruminants to the extreme environment of the Qinghai-Tibetan Plateau. In addition, ruminant host-specific taxa (*B. licheniformis*, *E. pyruvativorans*, and *L. bovis* in yak; *E. rhusiopathiae*, *R. bromii*, and *S. bovis* in cattle) that carry both ARGs and CAZymes were observed in each ruminant species under the same environment. This may be due to the host specificity of microbiome assembly [87], as well as divergent responses of yak and cattle to the environmental stress, since yak has adapted to high-altitude living conditions for a long time in terms of host’s genome [88] and rumen microbiome [11]. These findings suggest that various functions of the rumen microbiota could help the host to adapt to unstable environments, and this is partially driven by the host. The stability of the rumen microbiome was associated with animals’ health and nutrition [6, 7], thus, it is unsurprising that CAZymes and ARGs affect the microbial stability as they provide the host with many carbohydrate-active enzymes to digest forage polysaccharides efficiently and ARGs are associated with host health bacterial survival and some of these bacterial are the key fiber degraders. These microbial factors should be taken into consideration when exploring the microbial stability considering the importance of microbiome stability to the host.

**Fig. 11:**
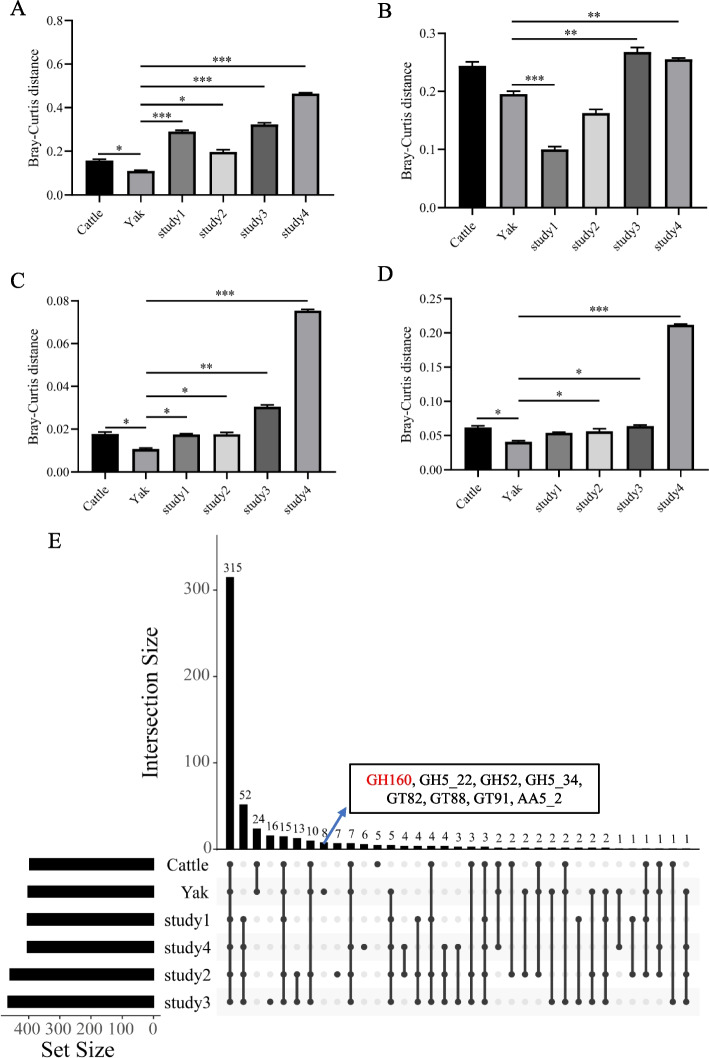
The dynamic changes of rumen microbial community and function among different studies. **A** Bacterial community similarity between the current study (cattle and yak) and other studies. **B** Archaeal community similarity between the current study (cattle and yak) and other studies. **C** KEGG pathways similarity between the current study (cattle and yak) and other studies. **D** CAZyme family similarity between the current study (cattle and yak) and other studies. **E** The distributions of CAZyme families in different studies. Studies 1–4 indicates that the published studies (* 0.05 < *P* < 0.01, ** 0.01 < *P* < 0.001, *** *P* < 0.001)

To validate our observed difference in the diversity and stability between yak and cattle rumen microbiomes, we further conduct the comparison using the metagenome datasets from grazing and feedlot cattle that are available in the public database [89–92]. Our observed higher rumen bacterial and archaeal diversity (Shannon index) in yak were confirmed (Additional file 2: Fig. S6). Moreover, the similarity of rumen bacterial and archaeal community and function (KEGG and CAZymes) (Fig. 11A-D) in yak was greater compared to that all cattle species [89, 91, 92]. These findings highlight that the rumen microbiome of yak probably possesses greater potential for nutrient utilization considering greater microbial diversity and similarity associated with high rate of dietary fiber fermentation [27] and capability for nutrients metabolism [93], respectively. Compared to previous studies (dairy cows and beef cattle) [90–92], the relative abundances of *Blautia producta*, *Parabacteroides distasonis*, and *Bacteroides cellulosilyticus* were higher in yak (Additional file 2: Fig. S7). *Blautia producta* could enhance the microbial adaptation to adverse environments via obtaining antibiotic resistance genes [94] and *Parabacteroides distasonis* promotes gut digestibility and absorption capacity through maintaining the intestinal barrier integrity [95]. In addition, *Bacteroides cellulosilyticus* is a cellulolytic bacterium [96]. Thus, the higher abundances of these microorganisms with beneficial functions in the current study might promote the ruminant’s adaptability to the harsh environment. In contrast, the relative abundances of *Ruminococcus bovis*, *Clostridium perfringens*, and *Clostridium botulinum* in the published studies [89–92] were greater than in both cattle and yak (Additional file 2: Fig. S7). *Ruminococcus bovis* is a species of amylolytic microorganisms [97] and members of *Clostridium* associated with gastrointestinal dysbiosis in dairy cows [98]. Metagenomics identified that KEGG pathways of carbohydrate metabolism, amino acid metabolism, and lipid metabolism were significantly enriched in yak compared to the published studies (Additional file 2: Fig. S8A), indicating that more efficient in utilization of these nutrients in the rumen of grazing cattle and yak. Moreover, the abundances of CAZyme families related to cellulose (GH6) and hemicellulose (GH3, GH51, GH78, and GH67) in this study were greater than the previous studies (Additional file 2: Fig. S8B), which promotes fiber degradation. In addition, GH160 was confirmed to be uniquely present in the rumen of grazing yaks when compared to the rumen microbiome from published studies (Fig. 11E), highlighting the potential for developing novel enzymes in the rumen of yak. Therefore, it is suggested that the rumen microbiome in the current study may have different rumen fermentation patterns and intestinal homeostasis, and yak rumen microbiome possess higher ability to utilize the low-quality forage when they were grazed on the Qinghai-Tibetan Plateau.

## Conclusions

In summary, metagenomics approach was employed to link the composition, function, and stability of the rumen microbiome of grazing ruminants raised under the harsh environment of the Qinghai-Tibetan Plateau. Our study revealed that rumen microbiome composition and function facilitated the adaptation of the grazing ruminants to this extreme environment. In this context, rumen microbes involved in fiber utilization (*F. succinogenes*, *R. albus*, and *T. thermophilus*) and methanogenesis (*M. mobile*) changed with the nutritional composition of the diet throughout the year. The rumen microbiome functional profiles (including amino acid biosynthesis, carbohydrate, and energy and CAZymes) also showed seasonal shifts to reflect the nutritional requirements of the host. A higher adaptability of the microbial community to the harsh environment was detected in yak compared to cattle. For example, yak rumen microbiome possessed more energy metabolism pathways and higher cellulase activity than that of cattle in the winter, especially when only low-quality forages were available. We also identified genes encoding novel CAZyme families in Yak rumen microbiome who also harbor a less diverse ARG profile compared to that of cattle, and these two microbial functions can influence the rumen microbiome stability together with the environment (season) and host biological (ruminant species) factors. Future studies are needed to fully characterize the CAZyme gene families at protein level as well as the ARG expression in the bacterial species that harbor these CAZyme gene families, and to evaluate their active effects on rumen microbiome stability among different ruminant species. The findings in the presence of unique CAZYmes and higher stability of the rumen microbiome of Yaks were further confirmed through the comparison with metagenomes of dairy and beef cattle, highlighting that yak rumen microbiota likely possesses greater potential for major nutritional metabolism (carbohydrate, amino acid, and lipid) and lignocellulose degradation compared with the rumen microbiome of beef cattle and dairy cows. The current study provides fundamental information about how the stability of the rumen microbiome changes throughout the year and describes microbial factors that may potentially contribute to this stability, as well as corroborate the point that yak rumen microbiome possesses higher ability to utilize the poor forage available compared to other ruminant species. Full understanding of the rumen microbiome functions paves the way for the development of technologies and practices that will contribute to improve productivity and food safety from ruminants globally.

## Methods

### Animal experiments, sample collection, and measurement of rumen fermentation parameters

Twelve castrated male ruminants (6 cattle (C1-C6); 6 yaks (Y1-Y6); body weight: 208 ± 19 kg; Age: 36 ± 4 month) were enrolled in this study. All animals were grazed together year-round at Wushaoling in Tianzhu Autonomous County, Gansu Province (37°12.4′N, 102°51.7′E; 3154 m altitude), China. Rumen fluid samples were collected via stomach tube before morning grazing at four different timepoints throughout a 10-month period, reflecting seasonal changes; spring (April), summer (August), fall (November), and winter (late January). Approximately 10 ml of each sample was used for pH measurement, and the remainder was immediately snap-frozen in liquid nitrogen and stored at -80 °C awaiting microbiome, NH_3_-N, VFA, and enzyme activity analysis [99–101]. Grass samples (alpine meadow) were also collected at the same time for feed chemical analysis [101]. Prior to VFA analysis, the frozen sample was thawed at room temperature and then centrifuged at 15,000 rpm at 4 °C for 15 min. 0.5 mL of the supernatant was then mixed with 0.1 mL of 25% metaphosphoric acid and VFA concentrations were determined using gas chromatography (SP-3420A, Beifenrili Analyzer Associates, Beijing, China) [102]. Ammonia N concentration of the rumen fluid was measured using the phenol-sodium hypochlorite colorimetry method [103]. To measure rumen microbial enzyme activity, the rumen fluid sample was thawed at room temperature followed by sonication in an ice water bath at a 30 s pulse rate for 10 min [104]. After centrifugation at 15,000 rpm at 4 ℃ for 20 min, the supernatant was used to evaluate the activity of cellulase, amylase, xylanase, and protease as previously described [100]. In brief, CMCase and xylanase activity were determined by measuring the release of reducing sugars from substrates (CMCase sodium and xylan, respectively, Sigma-Aldrich, St. Louis, MO, United States). The reaction mixture contained 0.5 ml substrate solution, 1 ml phosphate buffer and 0.5 ml collected supernatant, and the reaction lasted for 60 min (CMCase) and 15 min (xylanase) at 39 °C. For amylase activity, 0.25 ml starch solution was mixed with 0.5 ml phosphate buffer and 0.25 ml extracted supernatant and incubated for 30 min at 39 °C. The proteolytic activity was assessed by measuring the amount of azo dye after hydrolysis of azocasein (Sigma-Aldrich) in 1 h at 39 °C. The enzyme activities were expressed as umol of glucose and xylose released or hydrolysed protein per min per ml. For feed chemical analysis, the grass was dried at 65 °C for 24 h [104], and finely ground to pass through a 1 mm sieve for subsequent analysis. Neutral detergent fiber (NDF) and acid detergent fiber (ADF) fractions were measured as previously described [105] and were expressed with residual ash. Crude protein (CP) was measured using the Kjeldahl method [106]. Crude fat was extracted with petroleum ether according to the method described by Suárez et al. [107]. The ash content of samples was determined by combustion at 550 °C for 5 h.

### DNA extraction and metagenome sequencing

Frozen rumen samples were thawed and centrifuged at 12,000 g at 4 °C for 15 min, and the pellets were used for DNA extraction using the repeated bead-beating plus column purification method [108]. The quality and quantity of DNA was evaluated using a NanoDrop 2000 Spectrophotometer (NanoDrop Technologies, Wilmington, DE), and integrity was measured via visualization in a 1.0% agarose gel. Metagenomic DNA libraries were constructed using the TruSeq™ DNA Sample Prep Kit (Illumina, San Diego, CA). Following purification, each library was quantified using a Qubit 2.0 fluorimeter (Invitrogen, Carlsbad, CA, USA). Libraries underwent 2 × 150 nt paired-end sequencing on an Illumina Hiseq X Ten platform (Illumina, San Diego, CA) at Majorbio Bio-Pharm Technology Co., Ltd. (Shanghai, China).

### Data collection

The published rumen metagenomes (*n* = 127, Additional file 1: Table S13) available through NCBI SRA and the European Nucleotide Archive from the latest published literature were downloaded. The obtained sequences were published in 4 studies [89–92] comprising feedlot dairy cows, grazing dairy cows, and beef cattle (referred to as studies 1, 2, 3, and 4 in the text, Additional file 1: Table. S13). All collected sequences were analyzed with the data generated from this study together and using the analysis pipeline as follows.

### Bioinformatics analysis of sequencing data

FastQC (http://www.bioinformatics.babraham.ac.uk/projects/fastqc) was used to assess the quality of sequencing and downloaded data. Trimmomatic (v.0.38) [109] was used to remove short (< 50 nt) and poor quality (q < 25) reads, as well as Illumina sequencing adapters. The remaining high-quality reads were aligned to either the bovine (UMD 3.1.1) or yak (v1.1) genome to remove host DNA contamination using Bowtie2 [110]. Next, the trimmed and filtered reads were de novo assembled into contigs using Megahit (version 1.1.1) [111]. Taxonomic tags were assigned to contigs via alignment to a custom reference database using Kraken2 [112]. This database comprised all complete bacterial genomes deposited in RefSeq, as well as a further 913 rumen microbial genomes previously described [113]. Taxonomic abundance profiles at phylum, genus, and species levels were calculated, and only the taxa with relative abundance > 0.1% in at least 50% of the at least one timepoint within each species or in at least 50% of animals within each study were considered for further analysis. Alpha diversity (Shannon and Chao1) and beta diversity (Bray–Curtis dissimilarities) were calculated using species-level abundances. Alpha and Beta diversity measurements were calculated using the Vegan package in R [114].

For functional profiling, KEGG profiles were obtained using the MG-RAST platform [115] using previously described thresholds [116]. Contigs were annotated using DIAMOND v 0.9.31 [117] against the KEGG database [118] with an E value cutoff of 1e-5 to obtain the specific pathways information. Similarly, the ARG profile was determined via contig alignment against the CARD database (v.3.0.8) using DIAMOND with an E value less than 1e-5, percentage identity > 75% and bit score > 60 [78]. The CAZyme annotation was performed using USEARCH v8.1 [119] against the carbohydrate active enzyme database [120] with a minimum E value of 1e-5, bit score of > 60, and sequence identity of > 60%. Following annotation of CAZymes and ARGs genes, the rumen microbial origin of detected CAZymes and ARGs was identified by aligning the contigs encoding each CAZyme or ARG sequence to a customized reference database including genomes from metagenome-assembled genomes (MAGs) [5] using Kraken2. Abundances of ARGs, CAZyme and KEGG pathways in each sample were normalized into counts per million reads (CPM) for downstream analysis, and only those with a CPM > 1, in at least 50% of animals within each ruminant species in one season or in at least 50% of animals within each study were retained [121].

### Rumen microbiome stability analysis

Taxa-function robustness was calculated to determine the microbial stability, which assesses the degree to which a perturbation in a microbial community will influence its functional profile [122]. This data analysis was in accordance with a previous study [122], with slight modifications according to the author’s suggestions. Here, two indices, α (attenuation) and b (buffering), were used to directly assess the taxa-function robustness. Attenuation evaluates the expected rate at which increases in the magnitude of taxonomic perturbation are expected to influence the functional profile shifts of a microbial community, while buffering describes how large a perturbation must be before a functional profile shift becomes noticeable and approaches the expected shift predicted by attenuation [122].

### Statistical analysis

To assess the temporal variability of microbial composition within each ruminant species, the coefficient of variation (CV) was calculated, with higher values indicating higher temporal variation [123]. The diversity of taxonomic and functional profiles was determined using the ‘vegan’ package in R v3.5.3. Dissimilarity of the rumen microbial profiles across species was assessed using Analysis of Similarity (ANOSIM) with 999 permutations and visualized with Non-Metric Multidimensional Scaling (NMDS) ordination plots. The variation of rumen fermentation parameters (including enzyme activity, VFA, pH and Ammonia-N), chemical composition of natural pasture, and alpha and beta diversity indices, as well as their interactions were determined using mixed effects models implemented in the nlme R package [124]. Differences in fermentation profiles across seasons were identified using repeated measures ANOVA and the Tukey test for multiple comparisons, and *P* values < 0.05 were defined as statistically significant. Differences in CAZymes, KEGG pathways, ARGs, and rumen microbiota across seasons or studies were assessed using the non-parametric Kruskal–Wallis test in combination with Dunn’s post-doc test for multiple comparisons. Differences were considered statistically significant at a False Discovery Rate (FDR) value < 0.05 using the Benjamin-Hochberg algorithm [125]. The abundances of CAZymes, KEGG pathways, and ARGs between yak and cattle were tested using linear discriminant analysis effect size (LEfSe) [126], and a LDA score > 2 and *P* value < 0.05 were treated as significant differences. Spearman rank correlation coefficients were calculated using the Hmisc R package [127], and only ARGs or CAZymes showed positive or negative correlations with attenuation and buffering at the same time were considered for further analysis. Circos plots were generated using the circlize R package [128].

## Supplementary Information

Additional file 1: Table S1. Pasture’s chemical composition at different seasons (on dry matter basis). Table S2. Summary of sequence data generated from rumen samples of grazing yak (*n*=6) and cattle (*n*=6). Table S3. Rumen bacterial composition at the phylum level between grazing yak and cattle under different seasons. Table S4. Rumen bacterial composition at species level between grazing yak and cattle at different seasons. Table S5. Rumen archaeal composition at the phylum level between grazing yak and cattle under different seasons. Table S6. Rumen archaeal composition at species level between grazing yak and cattle at different seasons. Table S7. Composition of metabolic pathways based on the first-level and second-level functions in the KEGG. Table S8. Rumen CAZyme profiles between grazing yak and cattle at different seasons. Table S9. The origin of CAZymes in the species of ruminal bacteria. Table S10. ARGs profiles between grazing yak and cattle at different seasons. Table S11. The origin of ARGs in the species of rumen bacteria. Table S12. The information of rumen bacteria that carry both CAZymes and ARGs. Table S13. The rumen metagenomes analyzed in this study.

Additional file 2: Fig. S1. The dynamic changes of Bray-Curtis distance between yak (*n*=6) and cattle (*n*=6) at different seasons. (A) Bacterial community similarity among different seasons between yak and cattle. (B) Archaeal community similarity among different seasons between yak and cattle. Statistical analysis was determined using the non-parametric Kruskal-Wallis test in combination with Dunn’s post-doc test for multiple comparisons, and *P* values were corrected by Benjamin-Hochberg algorithm (* 0.05 < *P* < 0.01, ** 0.01 < *P* < 0.001, *** *P* < 0.001). Fig. S2. Non-metric multidimensional scaling (NMDS) analysis plot based on Bray–Curtis metrics showed fungal (A) and protozoal (B) community (at species level) of grazing yak and cattle at different seasons. (C) Fungal community similarity between cattle and yak. (D) Protozoal community similarity between cattle and yak. Fig. S3. Significantly different (*P* < 0.05) bacterial phyla between cattle and yak across seasons. Significantly different was assessed by non-parametric Kruskal-Wallis test in combination with Dunn’s post-doc test for multiple comparisons. Fig. S4. CAZyme profiles of yak and cattle. (A) Venn diagrams displaying overlap and unique CAZymes between yak and cattle. (B) Seasonal shift of CAZyme profiles in yak and cattle. Venn diagrams were generated using Venny 2.1 (https://bioinfogp.cnb.csic.es/tools/venny/). Fig. S5. Profiles of rumen microbiota origin of observed ARGs and CAZymes. (A) Venn diagrams showing overlap and unique rumen bacteria host both ARGs and CAZymes between yak and cattle. (B) Seasonal profile of rumen bacteria host both ARGs and CAZymes between yak and cattle. Venn diagrams were created by Venny 2.1 (https://bioinfogp.cnb.csic.es/tools/venny/), the taxonomic information of sequences (host both ARGs and CAZymes) were obtained by aligning the corresponding contigs to rumen metagenome-assembled genomes (MAGs) [5] using Kraken2 [113].  Fig. S6. Comparison of bacterial (A) and archaeal (B) alpha diversity indices between the current study (cattle and yak) and other studies. (* 0.05 < *P* < 0.01, ** 0.01 < *P* < 0.001, *** *P* < 0.001). Studies 1-4 indicates that the published studies. Fig. S7. Rumen bacterial species significantly differed in relative abundances between the current study (cattle and yak) and other studies. Studies 1-4 indicates that the published studies. Fig. S8. Rumen microbial functions (KEGG pathways: A; CAZyme families: B) that significantly differed in relative abundances between the current study (cattle and yak) and other studies. Studies 1-4 indicates that the published studies.

## Data Availability

Rumen metagenome sequences were deposited at the NCBI Sequence Read Archive (SRA) under the accession number PRJNA1161428. All data generated or analyzed during this study are included in this published article, its supplementary information files, and publicly available repositories.
