# Supplementary material for: Seasonal stability of the rumen microbiome contributes to the adaptation patterns to extreme environmental conditions in grazing yak and cattle
Source: BMC Biol. 2024 Oct 23;22:240. doi: 10.1186/s12915-024-02035-4 (PMC11515522; doi:10.1186/s12915-024-02035-4)
Supplement: Supplementary file 2 — Additional file 2: Fig. S1. The dynamic changes of Bray-Curtis distance between yak (n=6) and cattle (n=6) at different seasons. (A) Bacterial community similarity among different seasons between yak and cattle. (B) Archaeal community similarity among different seasons between yak and cattle. Statistical analysis was determined using the non-parametric Kruskal-Wallis test in combination with Dunn’s post-doc test for multiple comparisons, and P values were corrected by Benjamin-Hochberg algorithm (* 0.05 < P < 0.01, ** 0.01 < P < 0.001, *** P < 0.001). Fig. S2. Non-metric multidimensional scaling (NMDS) analysis plot based on Bray–Curtis metrics showed fungal (A) and protozoal (B) community (at species level) of grazing yak and cattle at different seasons. (C) Fungal community similarity between cattle and yak. (D) Protozoal community similarity between cattle and yak. Fig. S3. Significantly different (P < 0.05) bacterial phyla between cattle and yak across seasons. Significantly different was assessed by non-parametric Kruskal-Wallis test in combination with Dunn’s post-doc test for multiple comparisons. Fig. S4. CAZyme profiles of yak and cattle. (A) Venn diagrams displaying overlap and unique CAZymes between yak and cattle. (B) Seasonal shift of CAZyme profiles in yak and cattle. Venn diagrams were generated using Venny 2.1 (https://bioinfogp.cnb.csic.es/tools/venny/). Fig. S5. Profiles of rumen microbiota origin of observed ARGs and CAZymes. (A) Venn diagrams showing overlap and unique rumen bacteria host both ARGs and CAZymes between yak and cattle. (B) Seasonal profile of rumen bacteria host both ARGs and CAZymes between yak and cattle. Venn diagrams were created by Venny 2.1 (https://bioinfogp.cnb.csic.es/tools/venny/), the taxonomic information of sequences (host both ARGs and CAZymes) were obtained by aligning the corresponding contigs to rumen metagenome-assembled genomes (MAGs) [5] using Kraken2 [113]. Fig. S6. Comparison of bacterial (A) and archaeal (B) [file 12915_2024_2035_MOESM2_ESM.docx]

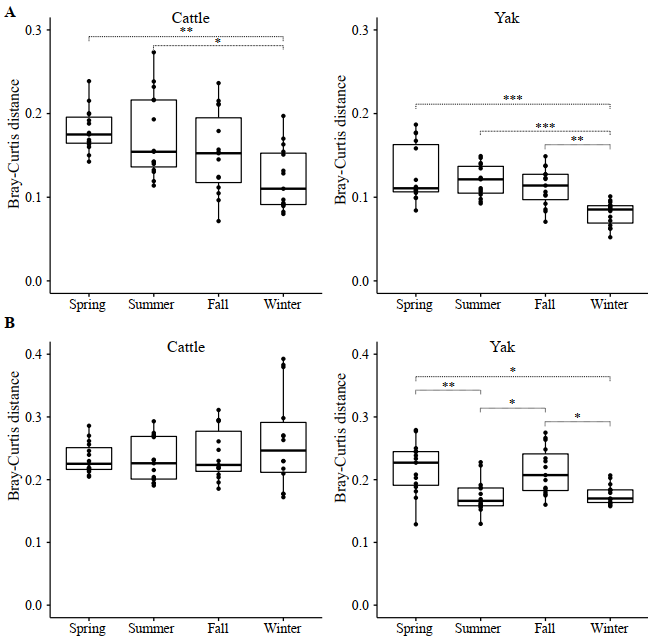


Fig. S1. The dynamic changes of Bray-Curtis distance between yak (n=6) and cattle (n=6) at different seasons. (A) Bacterial community similarity among different seasons between yak and cattle. (B) Archaeal community similarity among different seasons between yak and cattle. Statistical analysis was determined using the non-parametric Kruskal-Wallis test in combination with Dunn’s post-doc test for multiple comparisons, and *P* values were corrected by Benjamin-Hochberg algorithm (* 0.05 < *P* < 0.01, ** 0.01 < *P* < 0.001, *** *P* < 0.001).


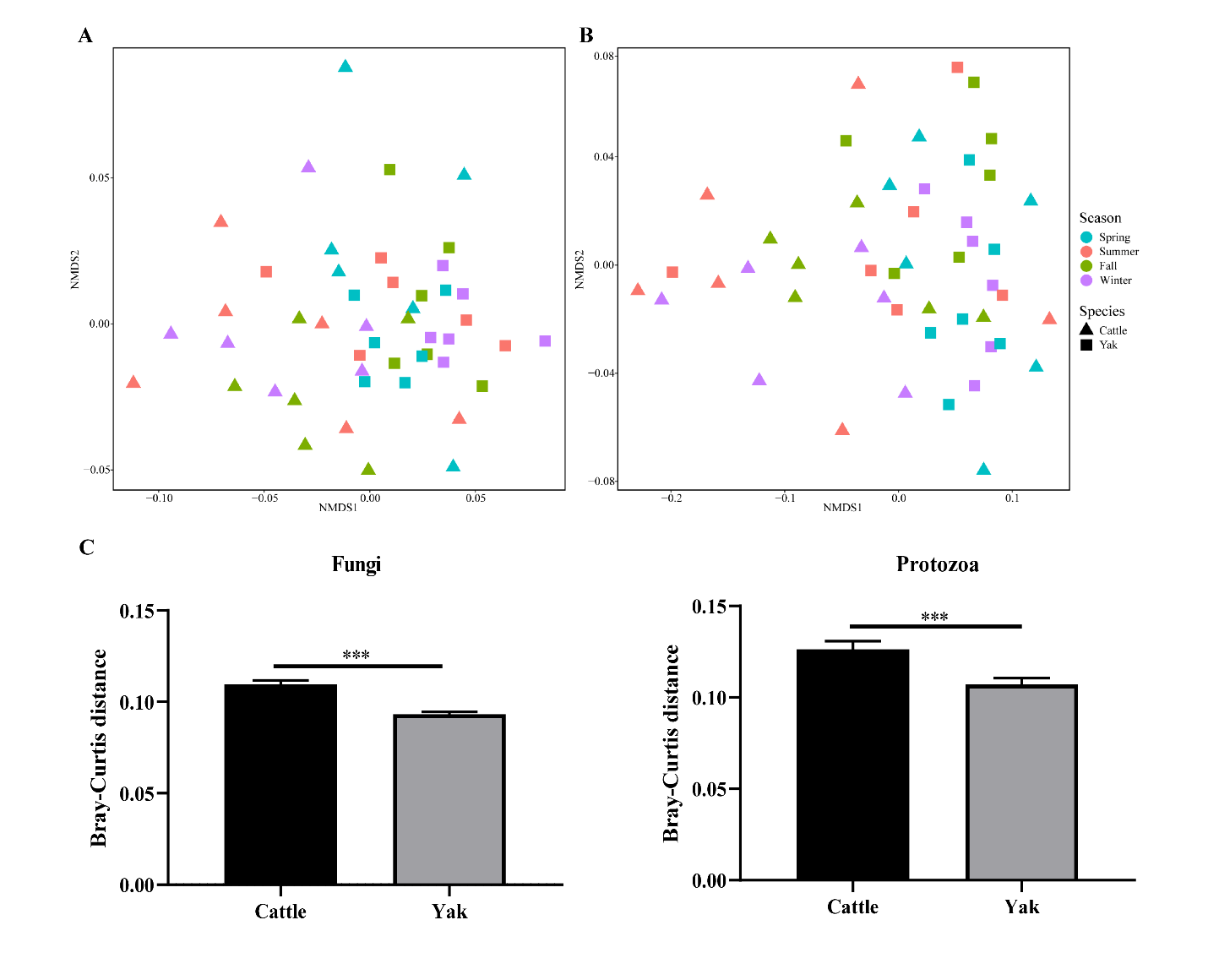


Fig. S2. Non-metric multidimensional scaling (NMDS) analysis plot based on Bray–Curtis metrics showed fungal (A) and protozoal (B) community (at species level) of grazing yak and cattle at different seasons. (C) Fungal community similarity between cattle and yak. (D) Protozoal community similarity between cattle and yak.


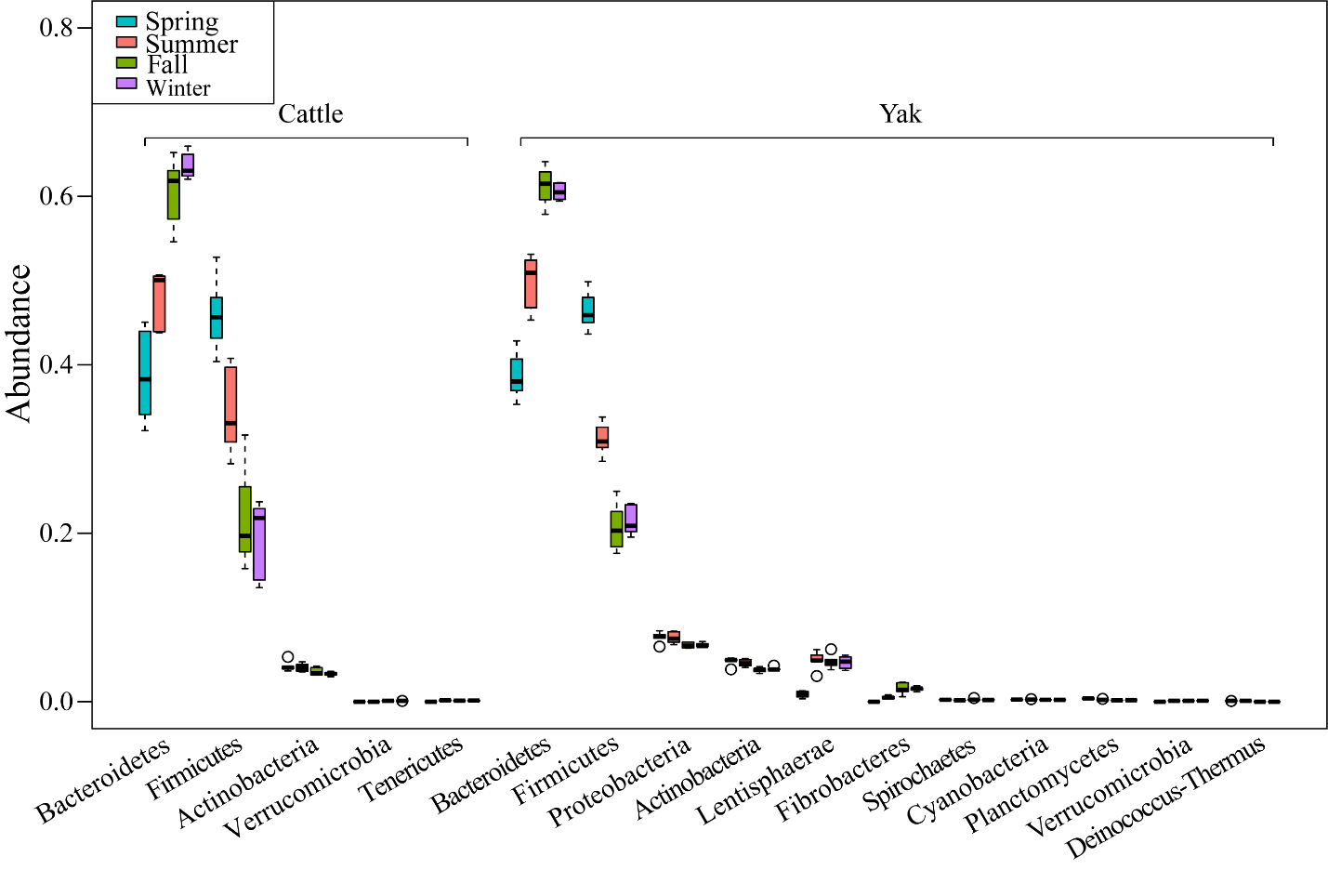


Fig. S3. Significantly different (*P* < 0.05) bacterial phyla between cattle and yak across seasons. Significantly different was assessed by non-parametric Kruskal-Wallis test in combination with Dunn’s post-doc test for multiple comparisons.


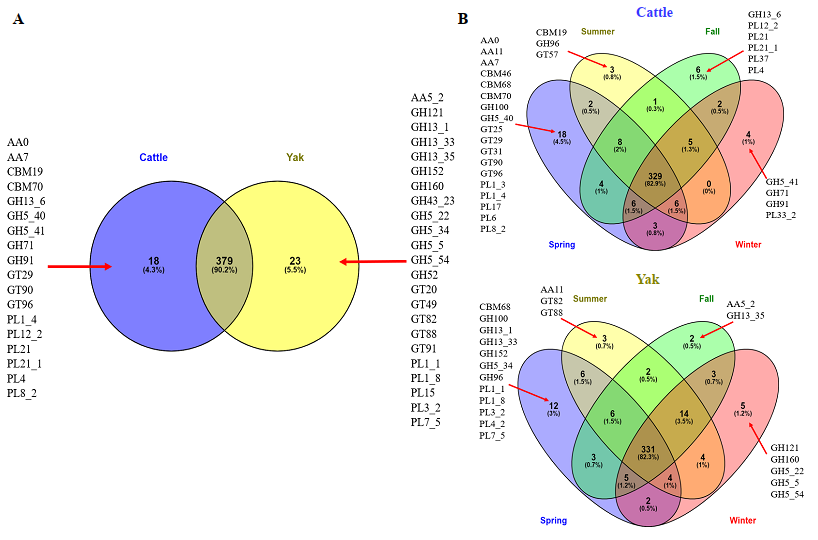


Fig. S4. CAZyme profiles of yak and cattle. (A) Venn diagrams displaying overlap and unique CAZymes between yak and cattle. (B) Seasonal shift of CAZyme profiles in yak and cattle. Venn diagrams were generated using Venny 2.1 (https://bioinfogp.cnb.csic.es/tools/venny/).


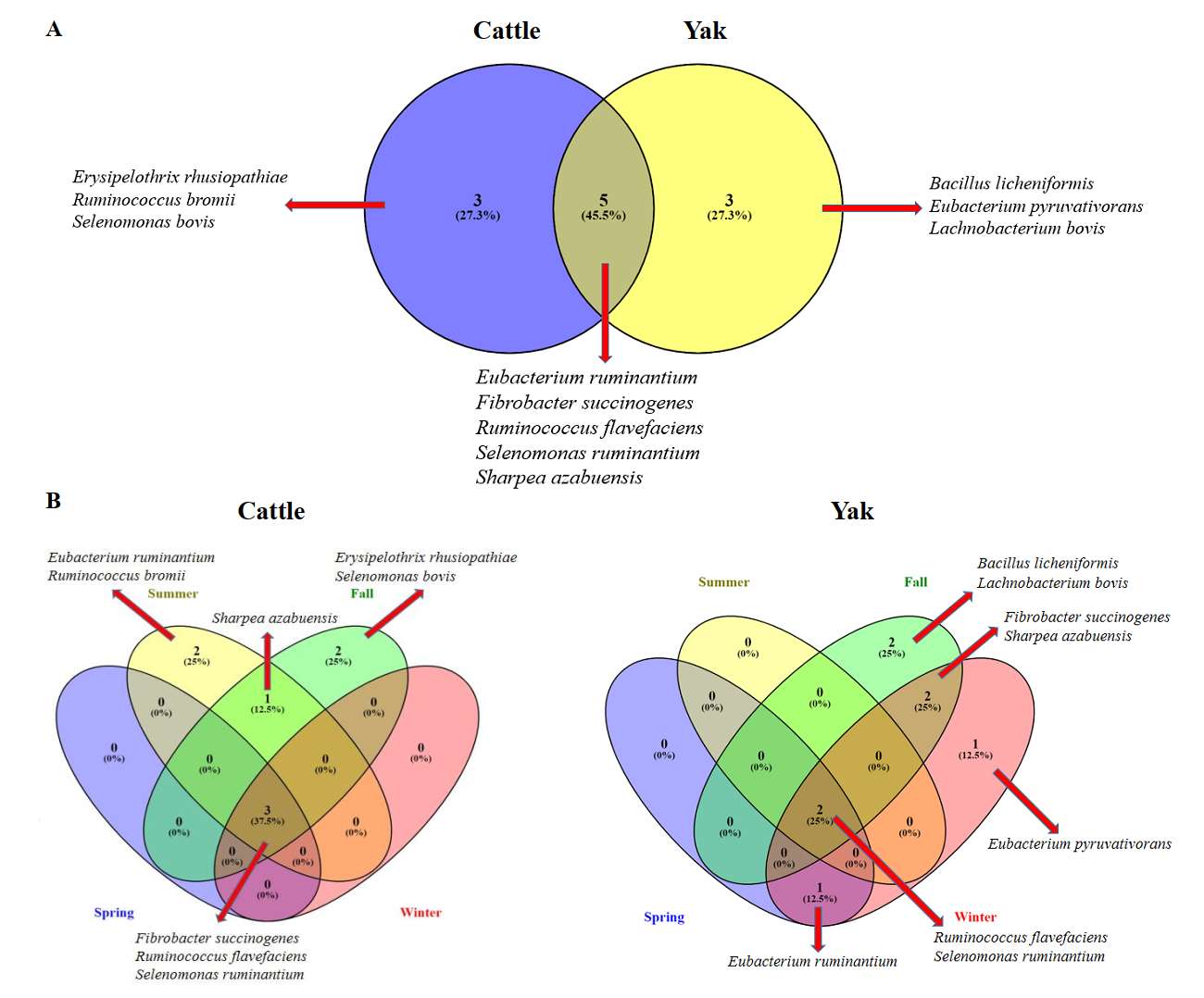


Fig. S5. Profiles of rumen microbiota origin of observed ARGs and CAZymes. (A) Venn diagrams showing overlap and unique rumen bacteria host both ARGs and CAZymes between yak and cattle. (B) Seasonal profile of rumen bacteria hosts both ARGs and CAZymes between yak and cattle. Venn diagrams were created by Venny 2.1 (https://bioinfogp.cnb.csic.es/tools/venny/), the taxonomic information of sequences (host both ARGs and CAZymes) were obtained by aligning the corresponding contigs to rumen metagenome-assembled genomes (MAGs) [5] using Kraken2 [112].


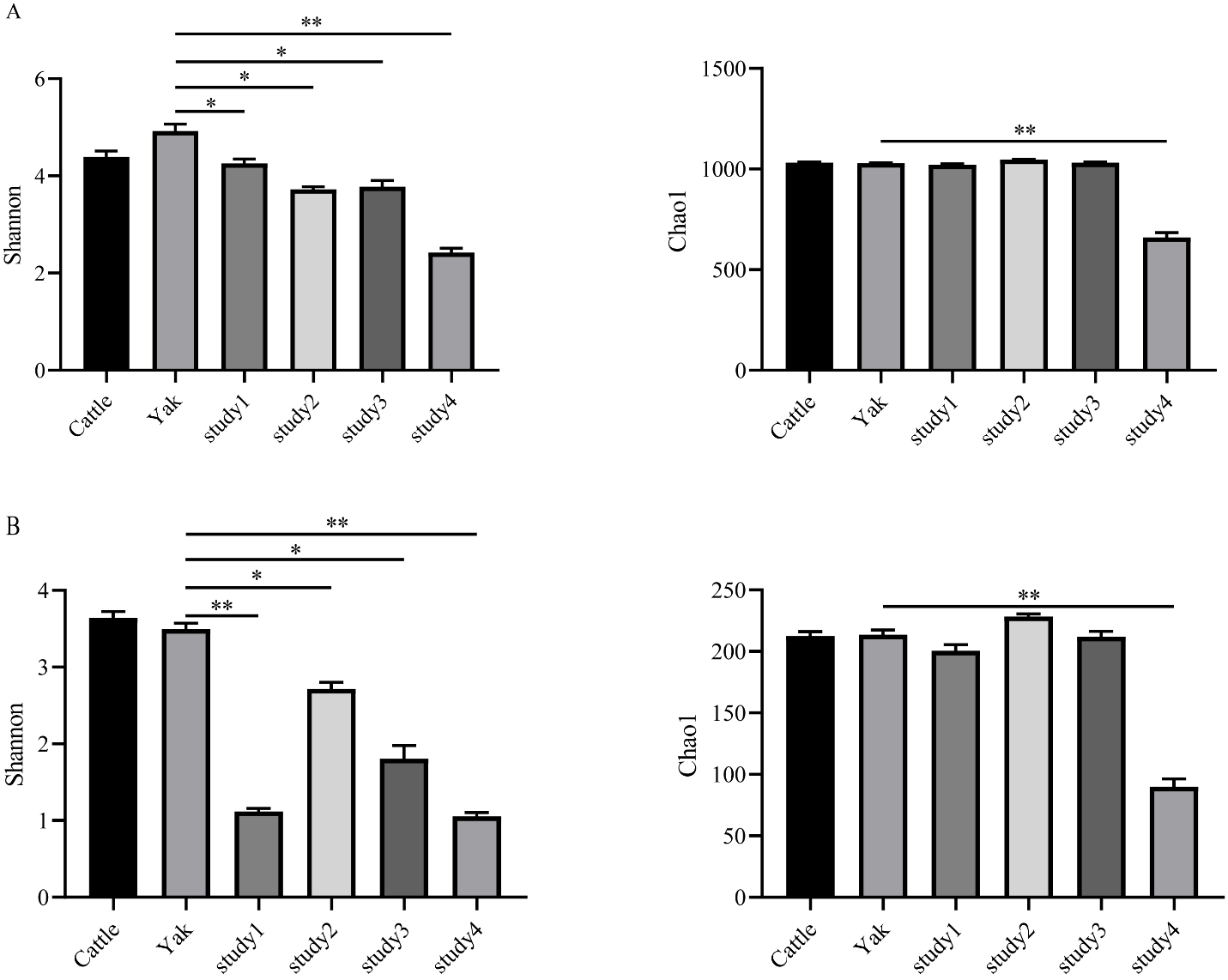


Fig. S6. Comparison of bacterial (A) and archaeal (B) alpha diversity indices between the current study (cattle and yak) and other studies. (* 0.05 < *P* < 0.01, ** 0.01 < *P* < 0.001, *** *P* < 0.001). Studies 1-4 indicates that the published studies.


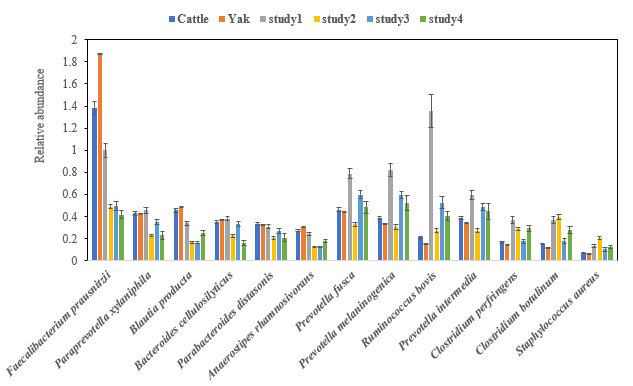


Fig. S7. Rumen bacterial species significantly differed in relative abundances between the current study (cattle and yak) and other studies. Studies 1-4 indicates that the published studies.


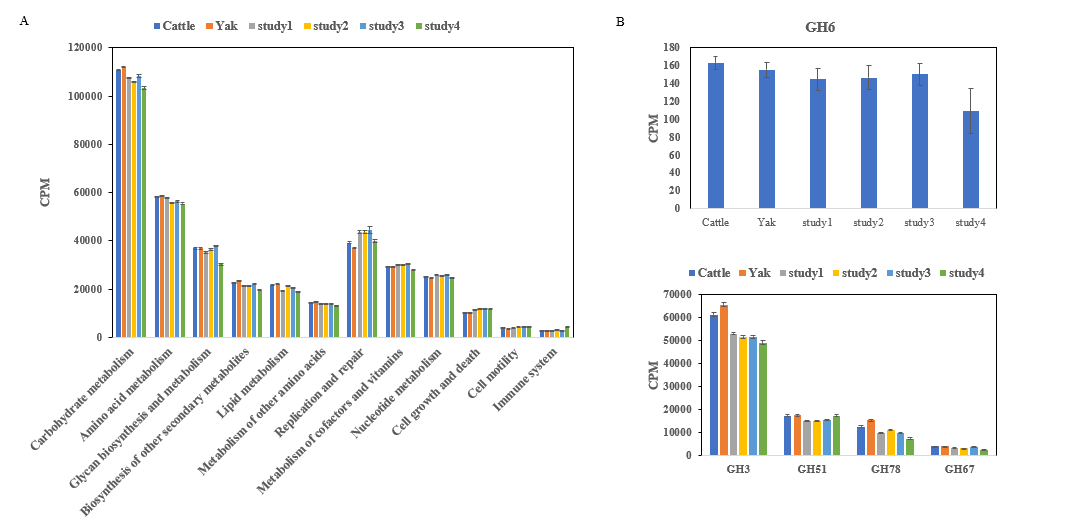


Fig. S8. Rumen microbial functions (KEGG pathways: A; CAZyme families: B) that significantly differed in relative abundances between the current study (cattle and yak) and other studies. Studies 1-4 indicates that the published studies.
